# Anti-inflammatory mechanisms and pharmacological actions of phycocyanobilin in a mouse model of experimental autoimmune encephalomyelitis: A therapeutic promise for multiple sclerosis

**DOI:** 10.3389/fimmu.2022.1036200

**Published:** 2022-11-03

**Authors:** Javier Marín-Prida, Nancy Pavón-Fuentes, Nielsen Lagumersindez-Denis, Hanlet Camacho-Rodríguez, Ana Margarita García-Soca, Rocío de la Caridad Sarduy-Chávez, Érica Leandro Marciano Vieira, Juliana Carvalho-Tavares, Viviana Falcón-Cama, Julio Raúl Fernández-Massó, Ignacio Hernández-González, Gillian Martínez-Donato, Gerardo Guillén-Nieto, Eduardo Pentón-Arias, Mauro Martins Teixeira, Giselle Pentón-Rol

**Affiliations:** ^1^ Center for Research and Biological Evaluations, Institute of Pharmacy and Food, University of Havana, Havana, Cuba; ^2^ Immunochemical Department, International Center for Neurological Restoration (CIREN), Havana, Cuba; ^3^ Institute of Neuropathology, University Medical Center Göttingen, Göttingen, Germany; ^4^ Biomedical Research Department, Center for Genetic Engineering and Biotechnology, Havana, Cuba; ^5^ Translational Psychoneuroimmunology Group, School of Medicine, Federal University of Minas Gerais (UFMG), Belo Horizonte, Brazil; ^6^ Department of Physiology and Biophysics, Institute of Biological Sciences, Federal University of Minas Gerais (UFMG), Belo Horizonte, Minas Gerais, Brazil; ^7^ Latin American School of Medicine (ELAM), Havana, Cuba; ^8^ Isotopes Center, San José de Las Lajas, Mayabeque, Cuba; ^9^ Laboratory of Immunopharmacology, Department of Biochemistry and Immunology, Instituto de Ciências Biológicas, Universidade Federal de Minas Gerais, Belo Horizonte, Minas Gerais, Brazil

**Keywords:** interferon-β, proinflammatory cytokines, remyelination, antioxidants, experimental autoimmune encephalomyelitis, multiple sclerosis, phycocyanobilin

## Abstract

Cytokines, demyelination and neuroaxonal degeneration in the central nervous system are pivotal elements implicated in the pathogenesis of multiple sclerosis (MS) and its nonclinical model of experimental autoimmune encephalomyelitis (EAE). Phycocyanobilin (PCB), a chromophore of the biliprotein C-Phycocyanin (C-PC) from *Spirulina platensis*, has antioxidant, immunoregulatory and anti-inflammatory effects in this disease, and it could complement the effect of other Disease Modifying Treatments (DMT), such as Interferon-β (IFN-β). Here, our main goal was to evaluate the potential PCB benefits and its mechanisms of action to counteract the chronic EAE in mice. MOG_35-55_-induced EAE was implemented in C57BL/6 female mice. Clinical signs, pro-inflammatory cytokines levels by ELISA, qPCR in the brain and immunohistochemistry using precursor/mature oligodendrocytes cells antibodies in the spinal cord, were assessed. PCB enhanced the neurological condition, and waned the brain concentrations of IL-17A and IL-6, pro-inflammatory cytokines, in a dose-dependent manner. A down- or up-regulating activity of PCB at 1 mg/kg was identified in the brain on three (*LINGO1*, *NOTCH1*, and *TNF-α*), and five genes (*MAL*, *CXCL12*, *MOG*, *OLIG1*, and *NKX2-2*), respectively. Interestingly, a reduction of demyelination, active microglia/macrophages density, and axonal damage was detected along with an increase in oligodendrocyte precursor cells and mature oligodendrocytes, when assessed the spinal cords of EAE mice that took up PCB. The studies *in vitro* in rodent encephalitogenic T cells and *in vivo* in the EAE mouse model with the PCB/IFN-β combination, showed an enhanced positive effect of this combined therapy. Overall, these results demonstrate the anti-inflammatory activity and the protective properties of PCB on the myelin and support its use with IFN-β as an improved DMT combination for MS.

## Introduction

The discovery of neuroprotective molecules that can promote remyelination in animal models has currently generated expectations for the development of new drugs for multiple sclerosis (MS). This is a central nervous system (CNS) disease that comprises chronic autoimmune-related demyelination and neurodegeneration producing sensorimotor dysfunction with high prevalence in young adults (1). MS has been classified according to its clinical presentation in: relapsing-remitting (RRMS), secondary progressive (SPMS), primary progressive (PPMS), and progressive relapsing MS (PR). Eighty percent of the patients with MS express the RRMS form which in turn, after 10-20 years, progresses to the SPMS form (2). Few disease-modifying therapies (DMT) have been found for the treatment of the main clinical forms (RRMS and SPMP) and even fewer for PPMS (3). MS pathogenesis has been associated with two processes, i.e. one related to the inflammatory response induced by a dysfunction of the immune system and the other related to the neurodegenerative events. DMTs that have received authorization for clinical use are directed against targets of the immune system, having little or no direct impact on neuroglial injury processes (4). Therefore, the development of drugs targeting myelin protection and/or remyelination or the use of combined therapies are relevant strategies in the therapeutic arsenal of this disease.

In this context, Phycocyanobilin (PCB), a linear tetrapyrrolic chromophore of C-Phycocyanin (C-PC) from the cyanobacterium *Spirulina platensis* could be a therapeutic candidate for MS. Our group has previously shown the beneficial effects of C-PC and PCB in nonclinical models of neurological diseases, such as the experimental autoimmune encephalomyelitis (EAE) model of MS (5–7) as well as rodent models of cerebral ischemia (8–10). Their effects were mediated by a diverse antioxidant, immunomodulatory, anti-inflammatory and myelin-protective/remyelinating capabilities (11).

In MS, the disruption of the blood-brain barrier (BBB) enables the cerebral infiltration of autoreactive T cells, increasing the levels of proinflammatory cytokines. As consequence, the molecular and cellular cascade initiated produces the CNS immune self-attack, which is most prominent at the first phases of MS in which the activated microglia and infiltrating macrophages destroy oligodendrocytes (OD) and promote demyelination (12). Indeed, the chief pathological feature of MS is the axonal demyelination and damage, which interrupts or decelerates the propagation of the action potential through the axons (13). The reverse process is the remyelination in which oligodendrocyte precursor cells (OPC) create new myelin sheaths on axons of the CNS. In the remyelination process, the neuronal functional recovery is fostered by either endogenous, through boosting neuronal repair processes, or exogenous, through stimulating OPC, the cells that produce myelin in the CNS (14). The remyelination is a very effective process in normal people and even at the early stages of MS, however it fails in the chronic stage of the disease (15), showing the importance of developing myelin-targeted therapies.

Hence, the objectives of our study were to understand the molecular and cellular mechanisms induced by PCB on the EAE inflammatory response and on the myelin protection and/or remyelination, specifically its effect on the activation, migration and differentiation of OPC. Furthermore, we investigated the potential benefit of the pharmacological combination of PCB with IFN-β (the first-ever approved DMT for MS) as a novel therapy for MS.

## Material and methods

### Reagents

The PCB (Cat. No. SC-396921, Santa Cruz Biotechnology, Inc., Dallas, USA) was solubilized with sterile PBS pH 7.4 at a concentration of 5 mg/mL. This stock solution was stored at -20 °C until use. When needed, a stock aliquot was diluted at the working concentration, preserved in dark, and immediately applied to the experiment. Unless otherwise indicated, every other reagent was acquired from Sigma-Aldrich (St. Louis, MO, USA) with its highest grade.

### Animals

This study used female C57BL/6 mice (8-10 weeks old) provided by the Animal Care Facilities of the Federal University of Minas Gerais, Belo Horizonte, Brazil. The standard husbandry conditions included food and water ad libitum and a light/dark cycle of 12 h. All national and international regulations for the procedures involving mice were followed, and the animal ethics committee of the Federal University of Minas Gerais approved this study protocol (No. CEUA - 255/2015).

### Establishment of EAE model

EAE was induced as previously described (16). Mice were subcutaneously (s.c.) injected with 0.1 mL emulsion, which contained 100 μg of murine MOG_35-55_ (CIGB, Havana, Cuba) diluted in 50 μL saline and 50 μL Freund’s Complete Adjuvant (CFA), and supplemented with 4.0 mg/mL of *Mycobacterium tuberculosis* H37RA (Difco Laboratories, Detroit, MI, USA), followed by two intraperitoneal (i.p.) administrations (day 0 and day 2 after immunization) of 300 ng pertussis toxin diluted in 200 μL saline. Control mice were treated with saline instead of the emulsion following a similar procedure. The neurological dysfunction was scored daily in a double-blind way using a modified scale (17). The score of 0 denoted no clinical signs, 0.5: tail tone reduction distally, 1: complete flaccid tail, 1.5: tail paralysis and walking imbalance, 2: tail paralysis and hind limb frailty (unilateral), 2.5: tail paralysis and hind limb frailty (bilateral), 3: hind limb paralysis (bilateral), 3.5: hind limb paralysis (bilateral) of and fore limbs frailty, 4: paralysis of hind and fore limbs (bilateral), and 5 indicated death. The area under the curve (AUC), representing clinical severity, was analyzed.

### Treatment schedules

Five experimental groups of mice were randomly distributed (n=6-7 per group). These included non-immunized animals (naïve), and EAE disease mice receiving the following i.p. treatments daily, once a day: sterile PBS (vehicle) or PCB at 0.1, 0.5, or 1 mg/kg, from day 0 until day 26 post-immunization. In follow-up experiments, animals (n = 9-10 each) received the vehicle or 1 mg/kg PCB daily by the oral route (intragastric gavage) once a day, or mouse 5000 U IFN-β (Cat. No. K00020, PBL, USA) subcutaneously (s.c.) every second day or the combination of both compounds. In these latest experiments, two schedules were assessed:

Prophylactic: PCB and IFN-β treatments were administered from day 0 of the induction to the end of the experiment (24 days)
Late therapeutic: PCB and IFN-β treatments were administered starting at 14 days post-induction until the end of the experiment (24 days)

### Histological and immunohistochemistry analysis of the mice spinal cords

Humane euthanasia was performed at the end of the study (day 26), followed by the perfusion with ice-cold PBS, and the fixation of the dissected spinal cords by immersion in 4% paraformaldehyde. The examined region of the spinal cord was the lumbar part, which is frequently and rapidly affected in EAE (18), and contains motor neurons that innervate the hindlimb muscles (19). The tissue was embedded in paraffin, and between 9-10 serial sections of 1-3 μm thickness were cut at 100 μm intervals in the rostral to caudal direction, from paraffin blocks using a sliding microtome and used for staining. Demyelination was evaluated by staining with Luxol fast blue combined with periodic acid-Schiff (LFB-PAS). The inflammatory markers were assessed by immunohistochemistry (IHC) with the following primary antibodies for T cells and macrophages/activated microglia, respectively: anti-CD3 (1:150, Cat. No. C1597C01, DCS, Hamburg, Germany) and anti-Mac-3 (CD107b) (1:200, Cat. No. 553322, BD Bioscience, Franklin Lakes NJ, USA). Additionally, an anti-amyloid precursor protein (APP) (1:2000, Cat. No. MAB348, Chemicon, Temecula, CA, USA) was used for evaluating the axonal injury. Moreover, an anti-tubulin polymerization-promoting protein (TPPP/p25) (1:500, Cat. No. 92305, Abcam, Cambridge, UK) and an anti-Olig2 (1:300, Cat. No. 18953, IBL, Hamburg, Germany) were used for detecting mature OD and OPC, respectively. A streptavidin-biotin protocol (biotinylated secondary antibodies and streptavidin conjugated to horseradish peroxidase) was performed to reveal the antigen-primary antibody binding with 3,3’-diaminobenzidine as the chromogen.

### Morphometric analysis

The stained spinal cord sections were photographed by using a digital camera (Software Cell Sens Dimension v.1.7.1, DP71, Olympus, Germany) coupled with a light microscope (Olympus BX51, Germany). The Image J software (v. 1.46r, NIH, USA) was used for the measurement of the areas and the number of marked cells. The percentage of demyelinated area, as well as the densities of immunostained cells for each marker (CD3, Mac-3, TPPP/p25, Olig2 and APP) were determined in relation to the total area of the white matter, and expressed as % or # cells^+^/mm^2^, respectively.

### Quantification of the brain levels of IL-17A, IL-6 and IL-10

After performing the animals’ euthanasia once anesthetized on day 26 post-immunization, both mice’s cerebral hemispheres were dissected and divided sagittally at 2.0 mm lateral from bregma. The cerebral tissue closest to the sagittal suture was mechanically homogenized (IKA Works Ultra-Turrax, USA) by mixing 100 mg of tissue in 1 mL of an ice-cold extraction solution (0.4 M NaCl, 0.05% Tween 20, 0.5% bovine serum albumin, 0.1 mM phenylmethylsulfonylfluoride, 0.1 mM benzetonium chloride, 10 mM EDTA and 20 KIU aprotinin, prepared in PBS pH 7.4). After centrifugation at 15,000 ×g for 10 min at 4°C, the supernatants were stored at −70°C until use. The assessment of IL-17A, IL-6, and IL-10 levels was accomplished with the adequate ELISA Kit (R&D Systems, Minneapolis, MN, USA), following the supplier’s protocols.

### Quantitative real-time PCR

The dissected cerebral tissues after concluding the study (day 26) were conserved in RNAlater (Ambion Inc., Applied Biosystems, Foster City, CA, USA), homogenized, and provided for RNA isolation by using TRIzol (Invitrogen, San Diego, CA, USA). The cDNA synthesis was performed with 1 μg of RNA (High-Capacity cDNA Archive Kit, Applied Biosystems, Foster City, CA, USA) and then used for Quantitative Real Time PCR (qPCR) in triplicate, in accordance with the Minimal Information for Publication of qPCR Experiments (MIQE) guidelines (20). The BLAST algorithm was implemented for designing the gene-specific primers (**Supplementary Table 1**), which were used at 300 nM (Metabion, Martinsried, Germany). The detection system consisted in the Fast SYBR Green PCR Master Mix and the Applied Biosystems 7900HT Fast Real-Time PCR System (Applied Biosystems, Foster City, CA, USA). Bi-distilled water, used as reaction control, did not generate any signal for either the target or the housekeeping genes. The 2-ΔΔCt method (21) implemented in the REST^©^ software (22) was used for the analysis and quantification of the mRNA levels.

### Measurement of Treg

The mice spleens were collected in HBSS under sterile conditions in a biosafety cabinet, minced with scissors, and passed through a steel mesh to homogenize the cell suspension containing red blood cells (RBCs). This was then subsequently lysed (9 parts of Milli-Q water and 1 part of sterile PBS 10X) and centrifuged (177× *g*, 10 min at 4°C). The resulting pellet was washed three times with ice-cold PBS 1X, and finally resuspended in RPMI-1640 medium supplemented with 10% fetal bovine serum (FBS) and 1% penicillin–streptomycin. The cell viability was assessed by the trypan blue technique and resulted above 95%. The single-cell suspensions were resuspended in the final concentration to 1 x 10^7^ cells/mL and marked for Fc receptor block (Innovex, NB309). After Fc block, the cells were co-incubated for 20 minutes at 4^°^C with cy-chrome (Cy), phycoerythrin (PE) or fluorescein isothiocyanate (FITC)-labeled antibodies that detect the cell surface markers CD4 or CD25, or with an anti-isotype control (eBioscience, and BD Pharmingen, San Diego, CA, USA). Thereafter, the cells were fixed with 2% formaldehyde, permeabilized and marked with a phycoerythrin (PE)-conjugated anti-Foxp3 monoclonal antibody by using the eBioscience™ Foxp3/Transcription Factor Staining Buffer Set (eBioscience, CA, USA). The appropriate controls (PE-labeled antibody and unstimulated cells) were incorporated in all assays. The signal acquisition was carried out with FACS CantoII (Becton & Dickinson, San Jose, CA, USA) above the 50 000 gated events on the lymphocytes population given the low frequency of positive events, and processed through the Diva^®^ software (Becton & Dickinson, San Jose, CA, USA).

### Analysis of the flow cytometry results

The analysis of the patterns and frequencies of CD4^+^ CD25^high^ T lymphocytes was performed with the FlowJo program (Tree Star, Ashland, OR, USA). Three different fluorochromes were used for each set of analyses, for example, anti-CD25-FITC, anti-Foxp3-PE, and anti-CD4-PE-Cy5.5 for Treg cells. The lymphocytes gate was selected, encompassing the upper right region of the dot-plot in which the double-positive cells for CD25-FITC and CD4-PE-Cy5 were present, and a new dot-plot was generated for selected the cells positive for Foxp3-PE (23). The quadrant markers were always limited based on the negative populations, the isotype controls and the fluorescence minus one control (FMO).

### Proliferation assay

The cell line (T_MBP-GFP_) was cultured and stimulated as previously reported (24). This line was previously stablished following a technique in which rat CD4^+^ T lymphocytes specifically reactive to myelin basic protein (MBP), were retrovirally engineered to produce green fluorescent protein (GFP) (25). T_MBP-GFP_ cells (4 x 10^4^ cells/well, 96-well plates) were co-cultured with irradiated rat thymocytes (5000 rad, 10^6^ cells/well) in complete DMEM/1% rat serum with 10 µg/mL MBP, with either of the following experimental conditions: PCB alone at 50, 100 or 200 μg/mL, IFN-β alone at 10^3^ U/mL, and the combination of 10^3^ U/mL IFN-β + PCB at 50, 100 or 200 μg/mL. After 48 h of incubation, MTT (3-[4,5-dimethylthiazol-2-yl]-2,5-diphenyltetrazolium bromide) at 0.5 mg/mL was incorporated to the medium, and cultured for another 4 h. CD4^+^ T cells from the spleen and lymphonodes of 2D2 mice were seeded at 2 x 10^5^ cells/well in RPMI medium with 10% fetal calf serum (FCS) and co-stimulated with 2 µg/mL anti-CD3/anti-CD28 at 37°C, 5% CO_2_. Purified CD4^+^ T cells were incubated with 5 x 10^3^ U/mL IFN-β alone, PCB alone at 10, 50 or 250 μg/mL or together with 5 x 10^3^ U/mL IFN-β. Two days later, CD4+ T cells were split 1:2 by using the same medium containing IL-2 at 10 ng/mL of final concentration, and incubated again for one day. Then, the cells were cultured with MTT at 0.5 mg/mL (by adding to the medium) for additional 4 h. After that, the medium was discarded, the formazan crystals were dissolved in 150 µL/well of dimethyl sulfoxide (DMSO), and a microplate reader (Perkin Elmer, Victor 2) was used for measuring the absorbance at 570 nm.

### Intracellular localization of PCB

T_MBP-GFP_ cells (5 x 10^5^ cells/well) were seeded in 12-well plates together with irradiated rat thymocytes (5000 rad, 1.25 x 10^7^ cells/well) in complete DMEM/1% rat serum with 10 µg/mL MBP and 200 µg/µL PCB for 3 h. Immediately afterwards, the nuclear DNA of T cells was labeled with DAPI following a previously described procedure with minor changes (26). Briefly, T cells were collected in 15 mL tubes, centrifuged at 400 g, at room temperature for 3 min, washed with PBS 1X (0.5 mL) and fixed during 10 min at 4°C with 4% paraformaldehyde (0.5 mL). T cells were treated first with 0.2% Triton X-100 (0.5 mL) and later with 1 µg/mL DAPI (0.5 mL) at room temperature for 5 and 10 min, respectively, with three washes of PBS 1X (0.5 mL) between them and again after completing the DAPI staining. By using a cytospin device (800 rpm, 3 min), T cells were transferred to microscope slides and cover slips were mounted with Fluoromount-G. Images acquisition was performed with a confocal microscope (Leica) for DAPI (λex=359 nm, λem=461 nm) and PCB (λex=550 nm, λem=591-641 nm) fluorescence. The Fiji software was used for analysis (27).

### Statistical analysis

The statistical analysis was carried out with the GraphPad Prism software 6.0 (GraphPad Inc., CA, USA). Data are expressed as the mean ± S.E.M. Kolmogorov-Smirnov normality test were applied to all data sets. One-way-ANOVA followed by Newman-Keuls tests, or Kruskal-Wallis followed by Dunn´s tests for multiple comparisons between three or more groups were used when appropriate. Statistical comparison for qPCR data was done with the REST^©^ software. P values of p<0.05 (*), p<0.01 (**), and p<0.001 (***) were considered statistically significant.

## Results

### PCB delayed and attenuated the clinical signs progression of EAE mice

**Figure 1:**
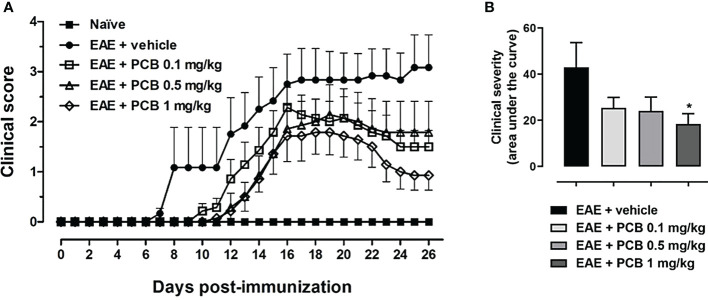
Effect of PCB in a murine model of chronic progressive EAE. **(A)** Daily clinical assessment of the mice. **(B)** Clinical severity determined by the area under the curve (in arbitrary units). MOG_35–55_ immunized mice received a daily i.p. injection of PBS (vehicle) or PCB at 0.1, 0.5 or 1 mg/kg, from day 0 until day 26 post-immunization. A numeric scale with the ascending graveness was used to score every day the clinical signs of the mice, ranging from 0 (no disease) to 5 (death). The naïve group (non-immunized animals) remained healthful during the entire study period (score 0). Data are expressed as the mean ± S.E.M (n=6-7 per group). The asterisk (*) is indicative of significant differences with vehicle-treated EAE mice (*p<0.05, ANOVA + Newman-Keuls tests).

The daily motor behavior of EAE mice receiving the intraperitoneal (i.p.) administration of either the vehicle or PCB at different doses was evaluated from the day of immunization until the end of the study (**Figure 1**). Interestingly, on average, the EAE + vehicle group presented the onset of the disease on day 9.8 ± 1.14, with the first animal being sick on day 7 following the immunization (**Figure 1A**). In contrast, the treatment with PCB at 0.1, 0.5 and 1 mg/kg postponed the beginning of clinical symptoms, on average, until the days 13.57 ± 1.82, 15.57 ± 1.81 and 15.29 ± 1.92 post-induction, respectively. Notably, the delay of the disease onset was statistically significant when the 0.5 and 1 mg/kg doses of PCB were compared with the vehicle-treated EAE mice (p<0.05, Kruskal-Wallis + Dunn’s multiple comparisons test, **Supplementary Table 2**). On the other hand, **Figure 1B** shows the quantification of the area under the curve of clinical progression, which represents the EAE neurological severity. EAE animals treated with the vehicle showed an area of 43.04 ± 10.65 (arbitrary units), while this parameter decreased to values of 25.46 ± 4.46, 24.14 ± 5.92 and 18.39 ± 4.55 for PCB at 0.1, 0.5 and 1 mg/kg, respectively. Statistically significant differences were observed between the EAE vehicle- and the EAE PCB-treated animals at the highest dose. This result indicates that the i.p. administration of PCB was able to retard the appearance of the disease’s symptoms and to reduce the EAE neurological severity.

### PCB reduces pro-inflammatory cytokines in the brain of mice with EAE

**Figure 2:**
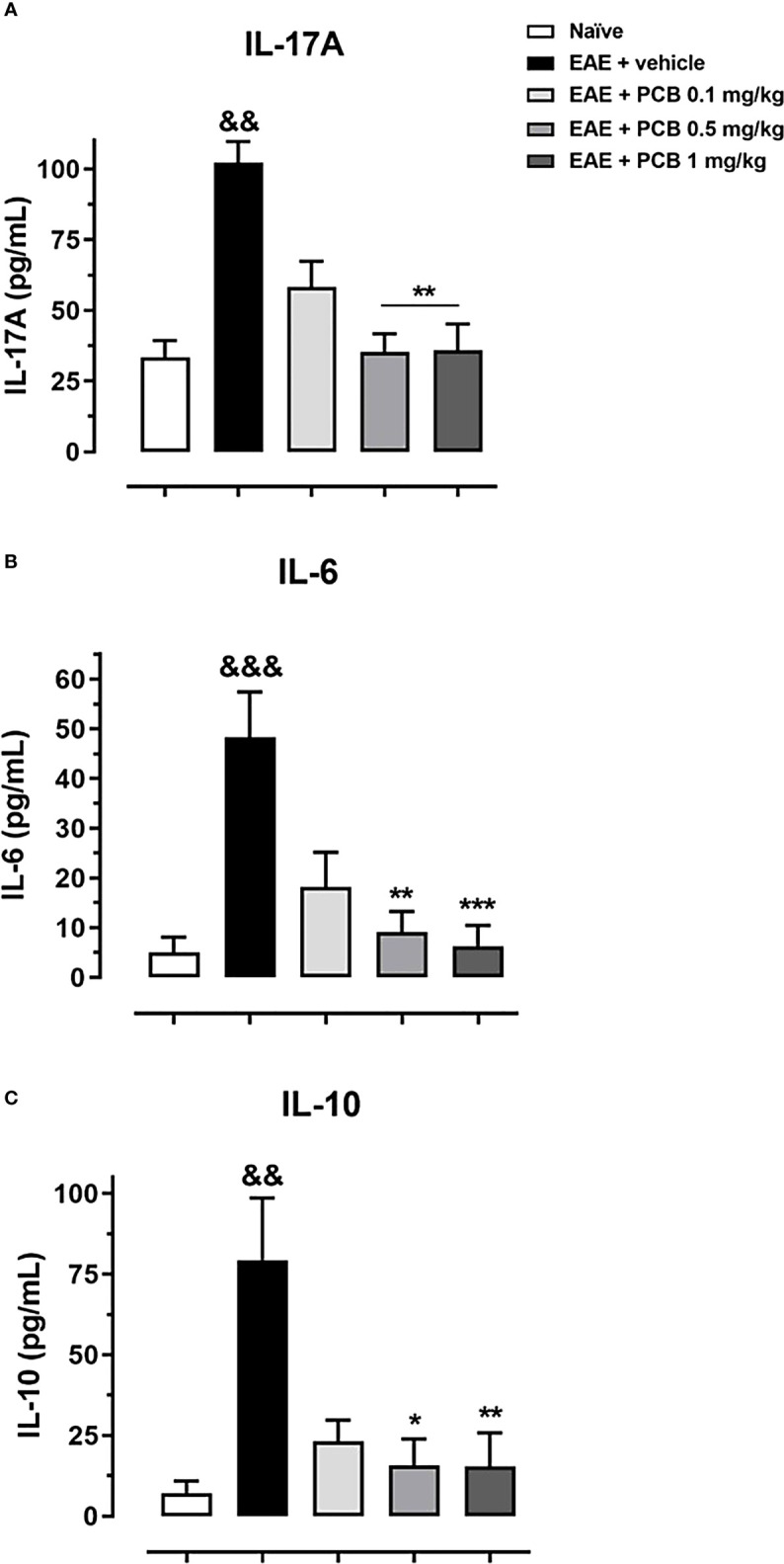
Evaluation of the PCB effect on the brain expression of cytokines in EAE mice. MOG_35–55_ immunized mice received a daily i.p. injection of PBS (vehicle) or PCB at 0.1, 0.5 or 1 mg/kg, from day 0 until day 26 post-immunization. At this moment, the protein levels of the cytokines **(A)** IL-17A, **(B)** IL-6, and **(C)** IL-10 were assessed by the respective ELISA commercial kits. Data are expressed as mean ± S.E.M. (n=6-7 per group). The ampersand (&) and asterisk (*) are indicative of significant differences with non-immunized naïve and vehicle-treated EAE mice, respectively (*p<0.05, **p<0.01, ***p<0.001; ^&&^p <0.01, ^&&&^p <0.001, Kruskal-Wallis + Dunn’s tests).

To further investigate the impact of PCB treatment on the expression of IL-17A, IL-6 (pro-inflammatory), and IL-10 (anti-inflammatory/immunoregulatory) cytokines in the brain of mice with EAE, an ELISA protocol has been used (**Figures 2A–C**). EAE vehicle-treated mice showed significantly higher levels of all measured cytokines compared to healthy naïve animals. Animals with EAE that received 0.5 and 1 mg/kg PCB evidenced a significant decline in the levels of IL-17A and IL-6 pro-inflammatory cytokines compared to the vehicle-treated EAE mice. These results suggest an anti-inflammatory action of PCB against the EAE progression in mice brains.

### PCB positively modulates the expression of genes related to remyelination/demyelination processes in mice brains

**Figure 3:**
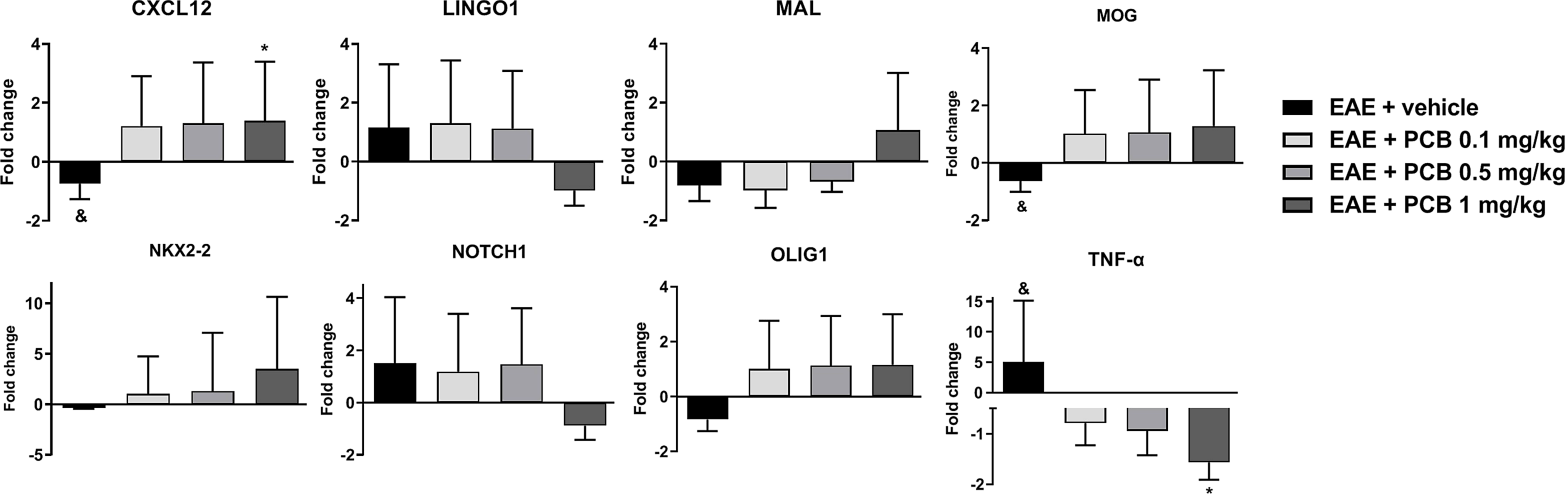
Effect of PCB on expression levels of demyelinating/remyelinating-related genes assessed by Real Time PCR in the brain of EAE mice. The panels show the mRNA levels of *CXCL12*, *LINGO1*, *MAL*, *MOG, NKX2-2, NOTCH1, OLIG1* and *TNF-α*. MOG_35–55_ immunized mice received a daily i.p. injection of PBS (vehicle) or PCB at 0.1, 0.5 or 1 mg/kg, from day 0 until day 26 post-immunization. Data are expressed as mean ± S.E.M. (n=4 per group). The ampersand (&) and asterisk (*) are indicative of significant differences with non-immunized naïve and vehicle-treated EAE mice, respectively (*p<0.05, ^&^p<0.05, REST^©^ software).

We evaluated the genes modulation in the brain of EAE animals treated with PCB at 0.1, 0.5 and 1 mg/kg (**Figure 3**). We observed an up-regulation of genes that could mediate myelin damage (*LINGO1*, *NOTCH1* and *TNF-α*), accompanied by the down-regulation of genes that may be involved in myelin preservation (*CXCL12*, *MAL*, *MOG*, *NKX2-2*, and *OLIG1*) in vehicle-treated EAE mice compared with naïve animals. In this ratio, a statistically significant difference was obtained for *TNF-α* (up), *CXCL12* (down) and *MOG* (down). The PCB-treated EAE animals at all doses, were compared to the EAE vehicle group. PCB at 0.1 mg/kg, regarding the genes that may influence the myelin destruction, only the *TNF-α* reversed its modulation (downward). On the contrary, all the myelin-favorable genes (except the *MAL* gene) inverted their modulation (upward), although no statistically significant differences were observed at this dose. When the sick mice received 0.5 mg/kg PCB, a similar modulation was detected in myelin-adverse genes. Finally, a dose-dependent effect of PCB was observed, which reached an inverse modulation for all genes studied at the highest dose of PCB (1 mg/kg). Moreover, a statistical difference was found for the *TNF-α* gene (down) and the *CXCL12* gene (up).

### PCB reduces demyelination and neuroinflammation in the spinal cords of EAE mice

**Figure 4:**
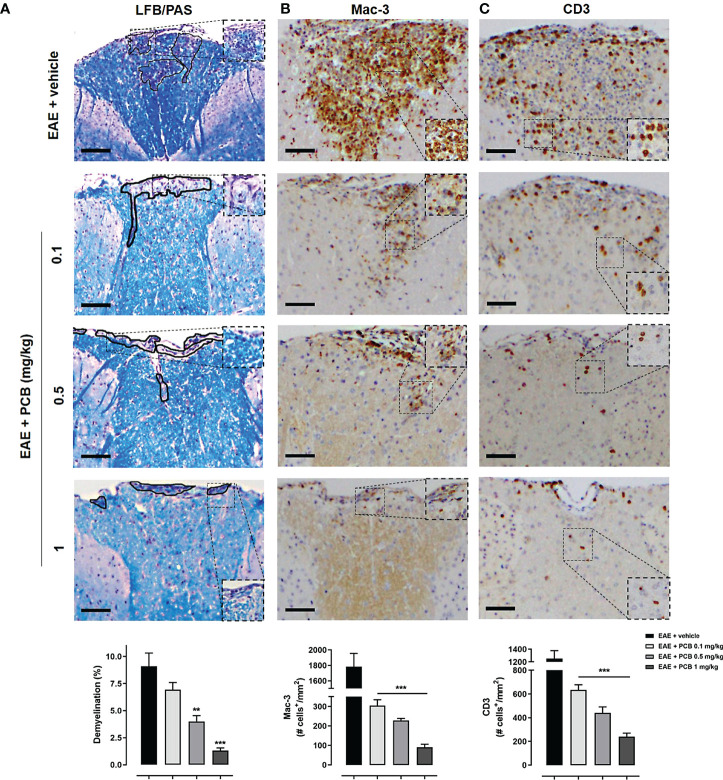
Histological and immunohistochemical assessment of the PCB effects on myelination and inflammation markers in spinal cords of EAE mice. The representative cross-sectional images and the respective morphometric evaluation per variable are shown in each panel. **(A)** Demyelination (LFB/PAS). **(B)** Macrophages/activated microglia (Mac-3). **(C)** T cells (CD3). MOG_35–55_ immunized mice received a daily i.p. injection of PBS (vehicle) or PCB at 0.1, 0.5 or 1 mg/kg, from day 0 until day 26 post-immunization. The areas marked with black pencil indicate demyelination. The percentage of demyelination and the cell densities (number of positive marked cells per mm^2^) were calculated by dividing with total area of the white matter in transversal sections. Data are expressed as mean ± SEM (n=4-7 per group). The asterisks indicate statistically significant differences vs. EAE + vehicle group (**p<0.01, ***p<0.001, Kruskal-Wallis + Dunn’s tests for LFB/PAS and Mac-3, ANOVA + Newman-Keuls tests for CD3). Bar: 50 μm.

Several areas of demyelination (purple area) were observed in EAE animals treated with the vehicle, also present (in a lesser proportion) with the lowest dose of PCB 0.1 mg/kg (**Figure 4A**). In contrast, intact myelin (expressed as an intense blue area) was observed in healthy mice (image not shown). Demyelination in the spinal cord white matter was clearly ameliorated in the diseased animals treated with all doses of PCB, particularly those of 1 and 0.5 mg/kg (**Figure 4A**). These results express the dose-dependent effect of PCB in reducing demyelination. The quantification of the demyelinated areas resulted in a significant decrease in this parameter in the EAE animals treated with the highest doses of PCB, 0.5 and 1 mg/kg, compared to those treated with the vehicle. On the other hand, immunostaining for Mac-3, a microglial and macrophages marker, and for the T lymphocytes marker CD3, were also carried out. The results expressed a significant inhibitory effect of PCB on both markers (**Figures 4B, C**). Representative images show the high density of Mac-3 and CD3 expression (brown spots) in the EAE + vehicle, which is lower in the groups of EAE mice that received PCB. No immunostaining for Mac-3 or CD3 was detected in the spinal cords of healthy mice (image not shown). Statistical analyses confirmed a significant dose-dependent diminution of the densities of Mac-3 and CD3 in PCB-treated EAE animas at all doses compared to those receiving the vehicle.

### PCB increases OPC, matured OD and reduces axonal damage in the spinal cords of EAE mice

**Figure 5:**
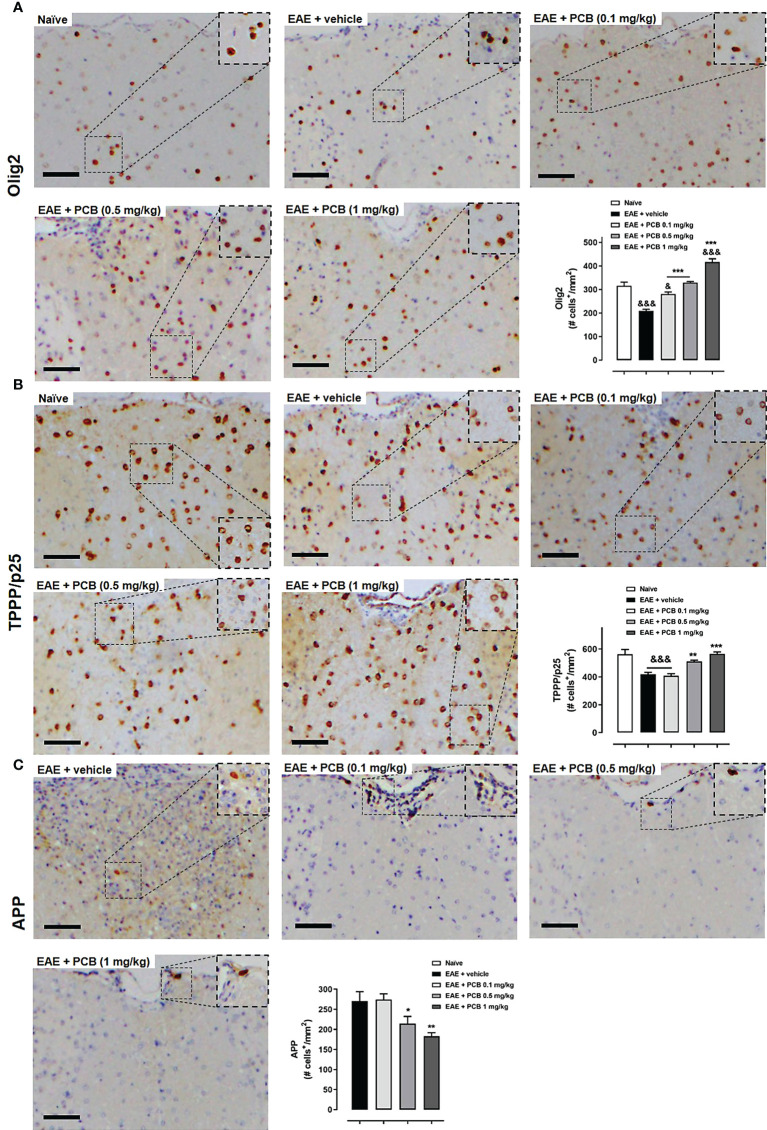
Immunohistochemical assessment of the PCB effects on oligodendrocyte and neuronal markers in spinal cords of EAE mice. The representative cross-sectional images and the respective morphometric evaluation per variable are shown in each panel. **(A)** Oligodendrocytes precursor cells (Olig2). **(B)** Mature oligodendrocytes (TPPP/p25). **(C)** Acute axonal damage (APP). MOG_35–55_ immunized mice received a daily i.p. injection of PBS (vehicle) or PCB at 0.1, 0.5 or 1 mg/kg, from day 0 until day 26 post-immunization. The cell densities (number of positive marked cells per mm^2^) were calculated by dividing with total area of the white matter in transversal sections. Data are expressed as mean ± SEM (n=4-7 per group). The ampersand (&) and asterisk (*) are indicative of significant differences with non-immunized naïve and vehicle-treated EAE mice, respectively (*p<0.05, **p<0.01, ***p<0.001; ^&^p<0.05, ^&&&^p<0.001, ANOVA + Newman-Keuls tests). Bar: 50 μm.

OPC and mature OD identification in the white matter of spinal cords was performed using an anti-Olig2 antibody (**Figure 5A**) or an anti-TPPP/p25 (**Figure 5B**), respectively. IHC was also performed for the APP as a marker of axonal damage (**Figure 5C**). A dose-dependent effect of PCB on the levels of these three markers was found when compared to the EAE animals treated with the vehicle. PCB at the highest dose (1 mg/kg) showed a statistically significant increase of both oligodendrocyte markers (Olig2 and TPPP/p25). Furthermore, there was a statistically significant rise of Olig2 in both the lowest and the intermediate doses of PCB (0.1 and 0.5 mg/kg). Regarding TPPP/p25, there was also an increase of this marker in the EAE + PCB 0.5 mg/kg group, but not at the dose of 0.1 mg/kg of PCB. Moreover, PCB also showed a dose-dependent effect on the APP density. At the intermediate and the highest dose of PCB, this treatment significantly reduced APP levels. However, there was no difference in APP density between the EAE mice treated with lowest doses of PCB compared to those treated with the vehicle (**Figure 5C**).

### “*In vitro*” and “*in vivo*” effects of PCB and its combination with IFN-β

The inhibitory effects of PCB and its combination with IFN- β was assessed in rat T_MBP-GFP_ cells and in T cells isolated from the spleen of 2D2 mice. There was a clear trend that PCB alone was able to inhibit the proliferation of MBP-stimulated rat T_MBP-GFP_ cells dose-dependently. However, when cultured with IFN-β alone at 10^3^ U/mL, no anti-proliferative action of this drug was observed. Remarkably, the combination of both compounds showed a statistically significant effect and the dose-dependent inhibition of T_MBP-GFP_ cell proliferation (**Supplementary Figure 1**). Furthermore, PCB also inhibited the proliferation of CD4^+^ T cells isolated from 2D2 mice. The results showed a clear tendency to reduction in the proliferation of these activated CD4^+^ T cells when treated with PCB alone at 10, 50 and 250 μg/mL (**Supplementary Figure 2**). However, this inhibitory effect was not evident with the treatment of 10^3^ U/mL IFN-β alone, but it was significant when combined with 250 μg/mL PCB (**Supplementary Figure 2**). On the other hand, the intracellular localization analysis revealed that PCB was accumulated in the perinuclear region of T_MBP-GFP_ cells (**Supplementary Video 1**).

**Figure 6:**
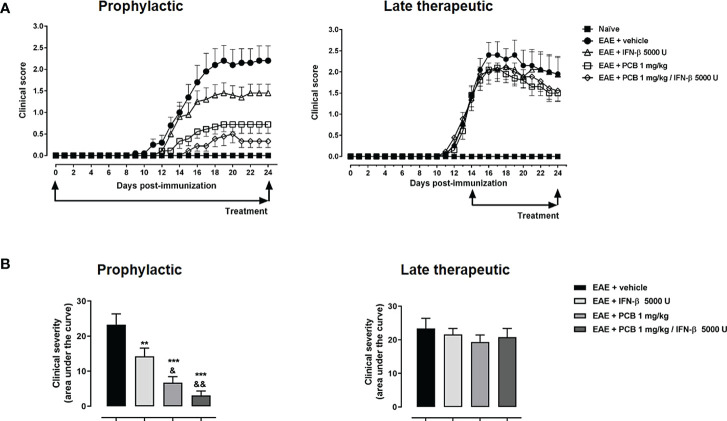
Effect of the treatment with the combination PCB/IFN-β and each compound individually on the clinical progression of EAE mice. **(A)** Daily clinical assessment of the mice. **(B)** Clinical severity determined by the area under the curve (in arbitrary units). MOG_35–55_ immunized mice were treated either with oral PCB at 1 mg/kg daily (once a day), with subcutaneous IFN-β at 5000 U every other day, or with their combination, initiating at day 0 (prophylactic schedule), or after the occurrence of the first disease symptoms at day 14 post-induction (late therapeutic schedule), lasting until day 24 post-immunization. A numeric scale with the ascending graveness was used to score every day the clinical signs of the mice, ranging from 0 (no disease) to 5 (death). The naïve group (non-immunized animals) remained healthful during the entire study period (score 0). Data are expressed as the mean ± S.E.M. (n=9-10 per group). The ampersand (&) and asterisk (*) are indicative of significant differences with IFN-β-treated or vehicle-treated EAE mice, respectively (**p<0.01, ***p<0.001, ^&^p<0.05, ^&&^p<0.01, ANOVA + Newman-Keuls tests).

**Figure 7:**
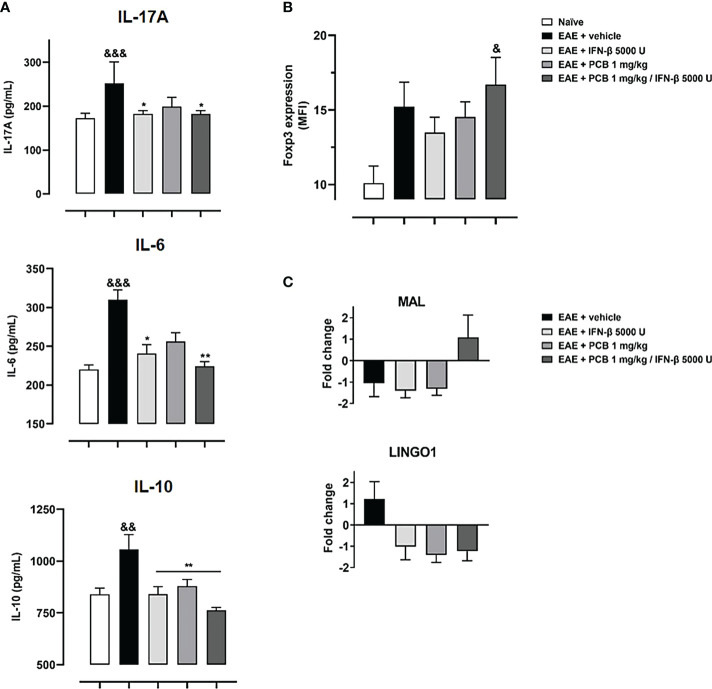
Effect of the treatment with the combination PCB/IFN-β and each compound individually on the cerebral expression of cytokines (protein levels) and of demyelinating/remyelinating-related genes (mRNA levels), as well as on the Treg levels in spleen of EAE mice. Panels show **(A)** the cytokines levels IL-17A, IL-6 and IL-10 from brain as assessed by ELISA, **(B)** Treg cells from mice spleen analyzed in the CD4^+^ CD25^high^ gate and expressed as Foxp3 Mean Fluorescence Intensity (MFI), **(C)** mRNA levels of *LINGO1* and *MAL* genes as assessed by quantitative Real Time PCR from the mice brain. MOG_35–55_ immunized mice were treated either with oral PCB at 1 mg/kg daily (once a day), with subcutaneous IFN-β at 5000 U every other day, or with their combination, initiating at day 0 (prophylactic schedule), or after the occurrence of the first disease symptoms at day 14 post-induction (late therapeutic schedule), lasting until day 24 post-immunization. Data are expressed as mean ± S.E.M. (n=4-7 per group). The ampersand (&) and asterisk (*) are indicative of significant differences in comparison to non-immunized naïve and to vehicle-treated EAE mice, respectively. (*p<0.05, **p<0.01, ^&^p<0.05, ^&&^p<0.01, ^&&&^p <0.001, Kruskal-Wallis + Dunn’s tests when compared with EAE + vehicle group for IL-17A and IL-16, or according to ANOVA + Newman-Keuls tests for IL-10 and MFI of Fopx3).

In the next set of experiments, we evaluated the PCB/IFN-β combination in the mouse model of EAE. When the prophylactic regimen was implemented, two EAE-treated groups (PCB and the combination) showed a significant decrease in the clinical severity as evidenced by the area under the curve of disease progression with respect to the EAE + vehicle group. However, when the administration was in the therapeutic schedule, starting at day 14 post-immunization once the disease symptoms were already present, no differences in the neurological progression between groups were found (**Figure 6**). Accordingly, when the expression of some cytokines were assessed after concluding the prophylactic regimen, the EAE vehicle-treated mice showed significantly higher expression of IL-17A, IL-6, and IL-10 when compared to the naïve group (**Figure 7A**). On the contrary, the EAE mice treated with the PCB/IFN-β combination significantly reduced the brain expression levels of these cytokines compared with EAE vehicle-treated mice, reaching levels comparable to those of naïve animals (**Figure 7A**). The individual treatments also significantly reduced IL-10, and for IL-17A and IL-6, only IFN-β was evident to be significant, although a clear decreasing trend was detected with PCB alone (**Figure 7A**). Interestingly, a significant induction of Treg was found, as evidenced by the mean fluorescence intensity in the spleen of animals treated with the PCB/IFN-β combination (**Figure 7B**). Regarding the gene modulation in the brain, we found that *LINGO1* and *MAL* genes were up- or down-regulated, respectively, in the EAE vehicle-treated versus untreated animals (**Figure 7C**). For the *MAL* gene, an inverse modulation (upward) was obtained only for the EAE animals treated with the PCB/IFN-β combination, but not with the separate treatments, compared to EAE vehicle-treated group (**Figure 7C**). However, the *LINGO1* gene remained down-regulated with either the individual or the combined treatments (**Figure 7C**).

## Discussion

In the present study, the outcomes and underlying mechanisms of the PCB administration were assessed in the EAE-MOG_35-55_ model of MS. We selected this model as appropriate for the PCB evaluation given the presence of both main components of the MS pathogenesis: immune dysfunction and neurodegeneration (5, 6, 11). Previously, we have reported that the anti-inflammatory and antioxidant properties of PCB and C-PC mediate their neuroprotective effects in animal models of cerebral ischemia (8–10). Such observations, therefore, suggest that these natural molecules might also curtail the pathogenesis of MS by acting on those pivotal deleterious processes.

Cytokines are mediators of both the innate and adaptive immunity (28). In this report, the administration of PCB in the MOG_35-55_ murine EAE model evidenced a significant dose-dependent reduction of cerebral expression of IL-17A and IL-6 in with respect to vehicle-treated EAE subjects. IL-17A and IL-6 are pro-inflammatory cytokines reported to play a key role in EAE (29). In this regard, several experimental models support the role of inflammatory cytokines in EAE; for example: anti-IL-17A antibodies cause a delay in disease progression in the EAE model (30), while the IL-17A blockade attenuates EAE (31), and mice either deficient of IL-17A or that received anti-IL-17A were protected from EAE establishment (32). In the same line, anti-IL-6 receptor monoclonal antibodies inhibited the development of EAE (33). Such evidence supports the positive impact of the cytokines reduction promoted by PCB in EAE. Moreover, we identified an increase in the IL-10 immunoregulatory cytokine in vehicle-treated EAE animals (similar to the IL-17A and IL-6 proinflammatory cytokines), which also decreased significantly with the PCB treatment. A possible explanation for this observation is that the immune system produces IL-10 as a negative feedback mechanism to maintain immune homeostasis from pro-inflammatory overreactions. Indeed, it has been shown that the IL-17 cytokine could also be produced not only by Th17 cells, but also by macrophages and T cells lacking either IL-10 or IL-10 receptor expression (34). The presence of recombinant IL-10 in the culture of these immune cells abrogates the production of IL-17, thus demonstrating the critical immune regulating role of IL-10 (34). Then, when the PCB treatment counteracts the overproduction of the pro-inflammatory cytokines (IL-17A and IL-6), the immunoregulatory component (IL-10) is also reduced as a balancing immune response.

Moreover, in our study, we inspected the dose-dependent outcome of PCB on the modulation of different genes related to the myelin physiology. We grouped them into genes whose products may injure the myelin in specific conditions (*LINGO1*, *NOTCH1*, and *TNF-a*) and those that could preserve it or participate in the myelogenesis multi-step process (*CXCL12*, *MAL*, *MOG*, *NKX2-2*, *OLIG1*).

The TNF-α cytokine has been shown to be a pleiotropic cytokine. In the cuprizone model of demyelination, animals deficient in TNF-α showed a significant delay in remyelination along with a significant decline in the number of mature OD (35). In contrast, the treatment of cuprizone-fed mice with XPro1595, a specific inhibitor of soluble TNF-α able to enter into the CNS, promoted early remyelination and impeded motor behavior deterioration (36), which suggests the relevant role of this cytokine in MS remyelination failure. That being so, it was also confirmed that the selective inhibition of soluble TNF-α improves recovery in the EAE mice model (37). In our study in diseased EAE animals (vehicle-treated), the *TNF-α* gene was up-regulated, however, EAE animals treated with the three doses of PCB showed down-regulation of the *TNF-α* gene. This suggests that PCB may favor the remyelination process by limiting the expression of TNF-α.

On the other hand, both the *LINGO1* and the *NOTCH1* genes, were up-regulated in the vehicle- or the PCB (0.1 and 0.5 mg/kg)-treated EAE animals, but both genes were down-regulated by the highest dose of this compound (1 mg/kg). It has been shown that the loss of the LINGO1 function in EAE mice led to a neurological improvement (38), while in the cuprizone animal model, an anti-LINGO1 antibody improved remyelination and neurobehavioral performance (39). Furthermore, the intranasal delivery of siRNA-loaded chitosan nanoparticles targeting LINGO1 was associated with signs of repair, neuroprotection, and remyelination in rats with ethidium bromide-triggered demyelination (40). On the other hand, by using the cuprizone animal model it was showed that inhibition of the *NOTCH1* gene expression by using an specific siRNA, accelerated remyelination mainly through increasing the mature OD in the brain lesions (41). In another study, with the aim of establishing whether NOTCH-JAGGED signaling regulated the rate of remyelination, young and adult animals were compared in the cuprizone model and no significant differences were found (42). Similarly, other authors have found that both NOTCH1 receptor and its ligand JAGGED1 were notably expressed in demyelinated areas of mice with EAE (43). Although the mechanistic role of LINGO1 and NOTCH1 in demyelination/remyelination processes in MS is yet to be fully elucidated, the available reports point to a deleterious action on the myelogenesis (44). Thus, the downregulation of these genes by PCB at the highest dose suggests a positive effect in promoting the formation of new myelin.

Furthermore, we identified the downregulation of the *CXCL12*, *MOG*, *NKX2-2*, *OLIG1* and *MAL* genes in the brain of sick EAE animals receiving the vehicle and an upregulation of the four first genes when those were treated with the three doses of PCB. The exception was found with the *MAL* gene in which its expression was increased only in the group EAE + PCB 1 mg/kg.

It has been observed that the lentivirus-mediated overexpression of CXCL12 in the *corpus callosum* with cuprizone-induced demyelination enhances OPC proliferation (45). Another study in which neonatal OPCs were isolated from rats showed that CXCL12 induces the activation of MEK/ERK and PI3K/AKT signaling in OPC promoting their migration (46). On the other hand, the primary physiological function of MOG, a protein exclusively present on the surface of myelin sheaths and OD processes, is not yet clearly established. However, its expression at the beginning of the myelination events and throughout the OD maturation process supports a likely involvement of this protein in the correct structural integrity of the myelin sheaths (47).

Other relevant protein in these aspects is NKX2-2, an important transcriptional factor for OPC differentiation. The expression of this gene has been detected in early CNS demyelinated lesions caused by ethidium bromide in rats, but not in old adult animals which have the slowest myelination speed. Thus, this evidence supports the notion that the abundance of NKX2-2 could accelerate remyelination in damaged CNS regions (48). In another study, by using *in situ* hybridization, immunofluorescence, and co-immunoprecipitation, it was demonstrated that the protein domains of NKX2-2 is critically present in OD differentiation (49). Similarly, another important mediator of myelin formation is OLIG1. It has been reported that when a neural progenitor cell (NPC) transplantation was performed in *OLIG1* ^-/-^ and *OLIG1* ^+/+^ mice infected with mouse hepatitis virus, only wild-type recipients exhibited extensive remyelination and differentiated preferentially in OPC (50). Previous reports have also suggested the potential involvement of OLIG1 in brain ischemia repair mechanisms when observed that its expression pattern was closely associated with endogenous remyelination (51). Besides these mediators, the MAL protein is predominantly expressed in OD, and it has been shown through genetic ablation procedures its relevant role in the axon-myelin interaction (52). Interestingly, a positive modulation of the *MAL* gene expression was also found in studies of our team using C-PC and PCB in animal models of MS (6) and cerebral ischemia (9), respectively. Therefore, the evidence shown above expresses the positive effects of PCB on these pro-myelinating genes and their potential involvement in myelin restoration in mice with EAE.

Accordingly, the LFB staining analysis of spinal cords revealed the presence of extensive demyelinated areas in mice of the EAE vehicle group, in agreement with previous reports (53, 54), as well as the significant increase of activated microglia, macrophages and T cells, supporting the inflammatory features involved in EAE (55, 56). In contrast, a significant reduction of CNS microglia/macrophages was observed in EAE animals that received any of the three PCB doses, and thus confirms its role in the action mechanisms for resisting this disease (57, 58). In the context of MS, activated microglia also contribute to neuronal and OD injury by acting as a paramount source of reactive oxygen species (ROS) (59). Indeed, microglial NADPH oxidase has been demonstrated to be one of the main sources of ROS in active MS lesions (60). The oxidative damage to lipids and DNA of the OPC, OD, and neurons, is closely associated with CNS demyelination and neurodegeneration (61). In this sense, PCB can inhibit the microglial NADPH oxidase (62). The inhibition of this enzyme is considerably beneficial in this context because it produces the superoxide anion radical which later reacts with nitric oxide favoring the rapid production of peroxynitrite, a key mediator of myelin and axon toxicity (63). PCB can also reduce oxidative damage by acting as a peroxynitrite scavenger (64). Therefore, the antioxidant activities of PCB may restore the redox balance in EAE mice and contribute to the myelin/axonal repair mechanisms.

Under pathological conditions, the activated microglia favors the infiltration of pro-inflammatory immune cells in the CNS by secreting matrix metalloproteinases (MMPs), mediators of BBB breakdown (65). Thus, the PCB treatment may prevent such events by limiting the rise in activated microglia. In addition, PCB can have a positive neuroaxonal effect by reducing the microglial secretion levels of pro-inflammatory cytokines, such as IFN-γ and TNF-α (66), and cytokines involved in the Th1 and Th17 response such as IL-12, IL-6 and IL-23 (67). The cytokines that mediate the inflammatory responses in MS and EAE may also directly impact on the myelinating capacity of OD (68). Indeed, IFN-γ and TNF-α act as pro-apoptotic inducers in human oligodendroglial cells, and furthermore, they potentiated the extent of cell death when co-incubated together (69).

In our study, an increase in OPC (identified by the Olig2 marker) and mature OD (evidenced by TPPP/p25) mediated the effects of PCB, suggesting the pro-myelination action of PCB, given that Olig2 functions as a transcription factor during the development of OD (70). Previously, it has been observed that the specific overexpression of Olig2 in OPC under demyelinating conditions promotes their migration to the CNS lesions and their differentiation into mature OD, which lead to an early remyelination process in lysophosphatidylcholine-intoxicated mice (71). Although this strongly suggests that PCB may induce the formation of new myelin in EAE mice, further evidence in this regard could be assessed in non-immunological demyelination models, such as the cuprizone or the lysophosphatidylcholine mice models.

The increase in the expression of Olig2 and TPPP/p25 caused by PCB indicates that this molecule favors the proliferative and migratory functions of OPC and their final differentiation into mature OD capable of myelinating the injured axons. The optimal expression of TPPP/p25 plays key physiological roles in the differentiation of OPC to mature myelinating OD (72). The TPPP/p25 protein is essential in the dynamics of the regeneration of the microtubule system during the elongation process previous to the myelin sheath structuring (73). These microtubules are essential for the structural stability and plasticity of myelinating OD. Furthermore, the evidence suggests that TPPP/p25 is also involved in the cellular metabolism, because its expression produces an increase in ATP levels (74). In MS patients, an increase in TPPP/p25 was detected in remyelinating lesions (73). OD that express TPPP/p25 extensively are able to form a compact myelin sheath that wraps the axon. The novel-formed myelin cover may physically curtail the direct action of immune cells and inflammatory molecules that mediate the damage to the axon, and it may also restore the trophic support to it (75).

Here, the axonal protecting activity of PCB was also evaluated, and its dose-dependent benefit at 0.5 and 1 mg/kg was demonstrated in animals with EAE. This could be related to the rapid restoration of the myelin trophic support that enables the survival and functionality of the axons. Therefore, it reduces axonal damage and the occurrence of intrinsic axonal defects that mediate physical and biochemical signals preventing myelination (76). The reduction of the abnormal accumulation of APP indicates that the axons are viable and rapid anterograde transport of APP occurs through them (77). The transported APP exerts trophic and synaptogenic roles, suggesting a restoration of the synapse (78), which is affected during demyelination (79).

On the other hand, *in vitro* studies in T_MBP-GFP_ encephalitogenic cells, demonstrated that PCB (and its combination with IFN- β) was able to inhibit the proliferation of these cells. Similarly, in these cells, the perinuclear location of the PCB was identified using the confocal microscope.

IFN-β is a standard treatment for MS, but it comes with limitations (80). In order to surpass this, the combination of IFN-β with other compounds acting on targets from either the immune or the nervous systems can potentially be a better therapeutic strategy for MS than IFN-β alone (81, 82). Hence, certain studies show the effects of IFN-β when co-administered with other DMTs. For example, one study showed a synergistic effect of IFN-β and dimethyl fumarate in EAE (83). A second study showed that the addition of a 1α,25-dihydroxyvitamin D3 analog with IFN-β produced additional immunomodulatory effects for the prevention of EAE (84). A third study demonstrated that the IFN-β-secreting mesenchymal stem cells and minocycline combination considerably reduced the clinical deterioration of EAE (85). A fourth study reported that the combined therapy of high doses of methylprednisolone with IFN-β had a synergistic effect in EAE (86). Our group demonstrated with a microarray assay of the brain of EAE mice that C-PC modulated additional desirable biological processes compared to IFN-β (6). Following this line of reasoning, here we report the beneficial effects of the PCB/IFN-β combination in EAE, thus supporting its complementarity.

The efficacy of IFN-β was assessed in the setting of EAE while combined therapies have also been tested in the EAE model demonstrating their effects (85, 87). In our study, the combined therapy of PCB/IFN-β, was, therefore, evaluated in the animal model of EAE. PCB in the combined therapy was administered orally, the efficacy of which had been previously demonstrated by our group when it was applied individually at different doses to EAE mice (7). This aspect is relevant for the potential clinical translation since the Spirulin-derived PCB administered orally has been issued the US Food and Drug Administration category of Generally Recognized As Safe (GRAS) (88).

A superior clinical effect was identified here in EAE animals treated with the PCB/IFN-β combined therapy when both molecules are administered prophylactically. However, no beneficial effect was observed when both molecules were administered in a late therapeutic regimen, which then open the perspective of a further evaluation with higher doses of these compounds. In our study, we have identified that both IFN-β and PCB, and to a greater extent, the treatment using both molecules, reduced the cerebral expression of pro-inflammatory cytokines IL-17A and IL-6. Consistently, the effectiveness of IFN-β in the EAE model induced by rodent recombinant MOG_35-55_ (independent of B cells) has been clearly related to the diminution of CNS pro-inflammatory cytokines (89). Another study also shows that the effectiveness of IFN- β is related to an inflammasome-dependent EAE model (90). Additionally, a greater induction of Treg in the spleen of the animals was achieved with the PCB/IFN-β combined therapy. A similar result on Treg was previously obtained by our group with the treatment of C-PC of peripheral blood mononuclear cells isolated from patients with MS (5). Along these lines of evidence, Treg can promote the differentiation of OPC and, consequently, favor their remyelination capability in the injured CNS (91). Interestingly, the PCB/IFN-β combined treatment differentially modulated the cerebral expression of *MAL* (upregulation) and *LINGO1* (downregulation) genes, supporting the remyelinating advantage of this pharmacological combination. PCB adds to this combination its anti-inflammatory, antioxidant, and remyelinating capabilities as have been shown in rodent models of EAE, and in other models of neurodegenerative diseases (92).

## Conclusion

There is an urgent need to identify MS drugs that target demyelination, which is a “gap” in the DMTs available thus far. PCB is well known for its neuroprotective and anti-inflammatory properties. In this study, we elucidated for the first time important clues of the mechanism by which PCB can promote remyelination in mice suffering EAE, comprising the stimulation of both the OPC and the mature OD. Moreover, PCB had also an advantageous impact on the reduction of axonal damage. On the other hand, the effect of PCB on the reduction of pro-inflammatory cytokines and its relationship with demyelination/remyelination processes is also highlighted in this study. Finally, the herein identification of the clinical superiority of the combination PCB/IFN-β, its associated reduction of pro-inflammatory cytokines and the increase of Treg, supports its potential application as a new feasible therapy for MS.

## Data availability statement

The original analysis of this study is presented in **Supplementary Table 2**. Inquiries regarding data sharing can be requested from the corresponding author.

## Ethics statement

The animal study was reviewed and approved by the institutional animal ethics committee of the Federal University of Minas Gerais, (CEUA - 255/2015), Belo Horizonte, Brazil.

## Author contributions

JM-P, conceptualization, writing, formal analysis, investigation, and funding acquisition. NP-F, formal analysis, investigation, and funding acquisition. NL-D, conceptualization, formal analysis, and investigation. HC-R, investigation (performing qPCR). AG-S, investigation (imaging and morphometry). RS-C, investigation (imaging and morphometry). ELMV, investigation and formal analysis (performance and analysis of flow cytometry). JC-T, investigation (EAE model) and writing. VF-C, investigation (microscopy analysis). JF-M, investigation, formal analysis, and methodology. IH-G, investigation and writing. GM-D, investigation and project administration. GG-N, methodology, project administration, and funding acquisition. EP-A, methodology and writing. MMT, methodology, writing, and funding acquisition. GP-R, conceptualization, writing, original draft, project administration, methodology, and funding acquisition. All authors contributed to the article and approved the submitted version.

## Funding

This work was partially supported by the Federal Ministry of Education and Research of Germany (BMBF) (Project number: 01DN18042) and the Science without Borders Program of Brazil (Project No. 405878/2013-3).

## Acknowledgments

We would like to thank Prof. Naoto Kawakami and Dr. Isabel Bauer for their technical assistance and contribution to the grant from BMBF.

## Conflict of interest

The authors declare that the research was conducted in the absence of any commercial or financial relationships that could be construed as a potential conflict of interest.

## Publisher’s note

All claims expressed in this article are solely those of the authors and do not necessarily represent those of their affiliated organizations, or those of the publisher, the editors and the reviewers. Any product that may be evaluated in this article, or claim that may be made by its manufacturer, is not guaranteed or endorsed by the publisher.

## Supplementary material

Supplementary Figure 1 Effects of the combination PCB + IFN-β on rat encephalitogenic T_MBP-GFP_ cells. Effect on the proliferation: T_MBP-GFP_ cells (4 x 10^4^ cells/well) were cultured for 48 h in different treatment conditions in 96-well plate with the presence of irradiated rat thymocytes (5000 rad, 10^6^ cells/well). Complete DMEM/1% rat serum with 10 µg/mL MBP was used. Immediately after, 0.5 mg/mL MTT was added to the medium and cultured for another 4 h. After medium removal, the resulting formazan was solubilized and its absorbance was measured at 570 nm. Data is presented as mean ± S.E.M. Ampersand (&) and asterisk (*) indicate significant differences in comparison to 10^3^ U/mL IFN-β and control, respectively (**p<0.01, ***p<0.001 vs control; ^&^p<0.05, ^&&^p <0.01 vs IFN-β, according to Kruskal-Wallis + Dunn’s tests).

Supplementary Figure 2 Effects of the combination PCB + IFN-β on the proliferation of CD4^+^ T cells from 2D2 mice. PCB was incubated with purified CD4^+^ T cells at different concentrations in the presence of absence of 5 x 10^3^ U/mL IFN-β. A seeding density of 2 x 10^5^ cells/well was used for the co-stimulation of CD4^+^ T cells with anti-CD3 and anti-CD28 at 2 µg/mL in 96-well plate. Two days later, CD4^+^ T cells were incubated with IL-2 at 10 ng/mL for another 24 h. Then, MTT assay was performed and its cellular reduction was measured by the absorbance at 570 nm. Positive and negative controls indicate the absence of the drugs in the medium or no incubation with anti-CD3/anti-CD28/IL-2/drugs, respectively. Data is presented as mean ± S.E.M. Ampersand (&) and asterisk (*) indicate significant differences in comparison to 10^3^ U/mL IFN-β and control, respectively (***p<0.001 vs control; ^&&^p <0.01 vs IFN-β, according to Kruskal-Wallis + Dunn’s tests).

Supplementary Video 1 T_MBP-GFP_ cells (5 x 10^5^ cells/well) were cultured in 12-well plate together with irradiated rat thymocytes (5000 rad, 1.25 x 10^7^ cells/well) in complete DMEM/1% rat serum with 10 µg/mL MBP and 200 µg/µL PCB for 3 h. Immediately after, nuclear DNA was labeled with 1 µg/mL DAPI, the image was taken by confocal microscopy and analyzed by Fiji software. Blue and red colors indicate the presence of the cell nucleus and PCB, respectively, in two T_MBP-GFP_ cells.
