# Supplementary material for: Anti-inflammatory mechanisms and pharmacological actions of phycocyanobilin in a mouse model of experimental autoimmune encephalomyelitis: A therapeutic promise for multiple sclerosis
Source: Front Immunol. 2022 Nov 3;13:1036200. doi: 10.3389/fimmu.2022.1036200 (PMC9669316; doi:10.3389/fimmu.2022.1036200)
Supplement: Supplementary file 1 [file Presentation_1.pptx]

## Slide 1
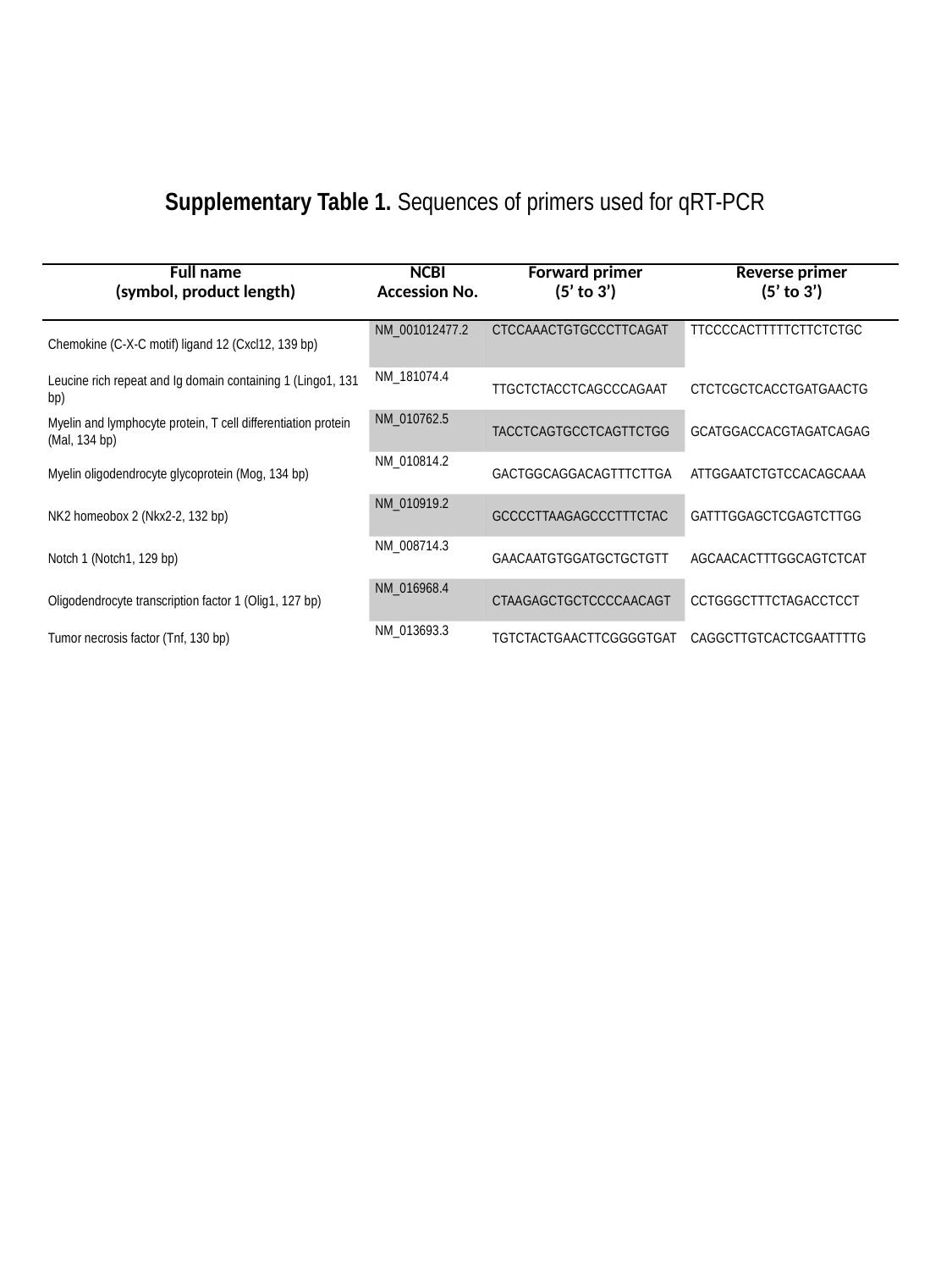

Supplementary Table 1. Sequences of primers used for qRT-PCR
| Full name (symbol, product length) | NCBI Accession No. | Forward primer (5’ to 3’) | Reverse primer (5’ to 3’) |
| --- | --- | --- | --- |
| Chemokine (C-X-C motif) ligand 12 (Cxcl12, 139 bp) | NM\_001012477.2 | CTCCAAACTGTGCCCTTCAGAT | TTCCCCACTTTTTCTTCTCTGC |
| Leucine rich repeat and Ig domain containing 1 (Lingo1, 131 bp) | NM\_181074.4 | TTGCTCTACCTCAGCCCAGAAT | CTCTCGCTCACCTGATGAACTG |
| Myelin and lymphocyte protein, T cell differentiation protein (Mal, 134 bp) | NM\_010762.5 | TACCTCAGTGCCTCAGTTCTGG | GCATGGACCACGTAGATCAGAG |
| Myelin oligodendrocyte glycoprotein (Mog, 134 bp) | NM\_010814.2 | GACTGGCAGGACAGTTTCTTGA | ATTGGAATCTGTCCACAGCAAA |
| NK2 homeobox 2 (Nkx2-2, 132 bp) | NM\_010919.2 | GCCCCTTAAGAGCCCTTTCTAC | GATTTGGAGCTCGAGTCTTGG |
| Notch 1 (Notch1, 129 bp) | NM\_008714.3 | GAACAATGTGGATGCTGCTGTT | AGCAACACTTTGGCAGTCTCAT |
| Oligodendrocyte transcription factor 1 (Olig1, 127 bp) | NM\_016968.4 | CTAAGAGCTGCTCCCCAACAGT | CCTGGGCTTTCTAGACCTCCT |
| Tumor necrosis factor (Tnf, 130 bp) | NM\_013693.3 | TGTCTACTGAACTTCGGGGTGAT | CAGGCTTGTCACTCGAATTTTG |
