# Supplementary material for: Anti-inflammatory mechanisms and pharmacological actions of phycocyanobilin in a mouse model of experimental autoimmune encephalomyelitis: A therapeutic promise for multiple sclerosis
Source: Front Immunol. 2022 Nov 3;13:1036200. doi: 10.3389/fimmu.2022.1036200 (PMC9669316; doi:10.3389/fimmu.2022.1036200)
Supplement: Supplementary file 2 [file Table_2.docx]

**Supplementary Table 2.** Statistical results obtained for each Figure by using the

GraphPad Prism software 6.0

| **Figure 1.** Effect of PCB in a murine model of chronic progressive EAE. | **Clinical severity (area under the curve)**   \| **One-way ANOVA** \| **SS** \| **DF** \| **MS** \| **F (DFn, DFd)** \| **P value** \| \| --- \| --- \| --- \| --- \| --- \| --- \| \| Treatment (between columns) \| 6246 \| 4 \| 1562 \| F (4, 29) = 6.878 \| P = 0.0005 \| \| Residual (within columns) \| 6584 \| 29 \| 227.0 \|  \|  \| \| Total \| 12830 \| 33 \|  \|  \|  \|  \| **Newman-Keuls multiple comparisons test** \| **Mean Diff.** \| **Significant?** \| **Summary** \| \| --- \| --- \| --- \| --- \| \| EAE + vehicle vs. EAE + PCB 0.1 mg/kg \| 17.58 \| No \| ns \| \| EAE + vehicle vs. EAE + PCB 0.5 mg/kg \| 18.90 \| No \| ns \| \| EAE + vehicle vs. EAE + PCB 1 mg/kg \| 24.65 \| Yes \| * \| \| EAE + vehicle vs. Naïve \| 43.04 \| Yes \| *** \| \| EAE + PCB 0.1 mg/kg vs. EAE + PCB 0.5 mg/kg \| 1.321 \| No \| ns \| \| EAE + PCB 0.1 mg/kg vs. EAE + PCB 1 mg/kg \| 7.071 \| No \| ns \| \| EAE + PCB 0.1 mg/kg vs. Naïve \| 25.46 \| Yes \| * \| \| EAE + PCB 0.5 mg/kg vs. EAE + PCB 1 mg/kg \| 5.750 \| No \| ns \| \| EAE + PCB 0.5 mg/kg vs. Naïve \| 24.14 \| Yes \| * \| \| EAE + PCB 1 mg/kg vs. Naïve \| 18.39 \| Yes \| * \|   **Day of the disease onset**   \| **Kruskal-Wallis test** \|  \| \| --- \| --- \| \| P value \| 0.0286 \| \| Exact or approximate P value? \| Approximate \| \| P value summary \| * \| \| Do the medians vary signif. (P < 0.05)? \| Yes \| \| Number of groups \| 4 \| \| Kruskal-Wallis statistic \| 9.052 \|  \| **Dunn's multiple comparisons test** \| **Mean rank diff.** \| **Significant?** \| **Summary** \| \| --- \| --- \| --- \| --- \| \| EAE + vehicle vs. EAE + PCB 0.1 mg/kg \| -5.833 \| No \| ns \| \| EAE + vehicle vs. EAE + PCB 0.5 mg/kg \| -11.98 \| Yes \| * \| \| EAE + vehicle vs. EAE + PCB 1 mg/kg \| -10.48 \| Yes \| * \| |
| --- | --- | --- | --- | --- | --- | --- | --- | --- | --- | --- | --- | --- | --- | --- | --- | --- | --- | --- | --- | --- | --- | --- | --- | --- | --- | --- | --- | --- | --- | --- | --- | --- | --- | --- | --- | --- | --- | --- | --- | --- | --- | --- | --- | --- | --- | --- | --- | --- | --- | --- | --- | --- | --- | --- | --- | --- | --- | --- | --- | --- | --- | --- | --- | --- | --- | --- | --- | --- | --- | --- | --- | --- | --- | --- | --- | --- | --- | --- | --- | --- | --- | --- | --- | --- | --- | --- | --- | --- | --- | --- | --- | --- | --- | --- | --- | --- | --- | --- | --- |
| **Figure 2.** Effect of PCB on cytokines levels in the brain of EAE mice | **IL-17A**   \| **Kruskal-Wallis test** \|  \| \| --- \| --- \| \| P value \| 0.0004 \| \| Exact or approximate P value? \| Approximate \| \| P value summary \| *** \| \| Do the medians vary signif. (P < 0.05)? \| Yes \| \| Number of groups \| 5 \| \| Kruskal-Wallis statistic \| 20.23 \|  \| **Dunn's multiple comparisons test** \| **Mean rank diff.** \| **Significant?** \| **Summary** \| \| --- \| --- \| --- \| --- \| \| Naïve vs. EAE + vehicle \| -25.76 \| Yes \| ** \| \| Naïve vs. EAE + PCB 0.1 mg/kg \| -10.27 \| No \| ns \| \| Naïve vs. EAE + PCB 0.5 mg/kg \| 0.000 \| No \| ns \| \| Naïve vs. EAE + PCB 1 mg/kg \| 1.450 \| No \| ns \| \| EAE + vehicle vs. EAE + PCB 0.1 mg/kg \| 15.49 \| No \| ns \| \| EAE + vehicle vs. EAE + PCB 0.5 mg/kg \| 25.76 \| Yes \| ** \| \| EAE + vehicle vs. EAE + PCB 1 mg/kg \| 27.21 \| Yes \| ** \| \| EAE + PCB 0.1 mg/kg vs. EAE + PCB 0.5 mg/kg \| 10.27 \| No \| ns \| \| EAE + PCB 0.1 mg/kg vs. EAE + PCB 1 mg/kg \| 11.72 \| No \| ns \| \| EAE + PCB 0.5 mg/kg vs. EAE + PCB 1 mg/kg \| 1.450 \| No \| ns \|   **IL-6**   \| **Kruskal-Wallis test** \|  \| \| --- \| --- \| \| P value \| 0.0003 \| \| Exact or approximate P value? \| Approximate \| \| P value summary \| *** \| \| Do the medians vary signif. (P < 0.05)? \| Yes \| \| Number of groups \| 5 \| \| Kruskal-Wallis statistic \| 21.24 \|  \| **Dunn's multiple comparisons test** \| **Mean rank diff.** \| **Significant?** \| **Summary** \| \| --- \| --- \| --- \| --- \| \| Naïve vs. EAE + vehicle \| -28.85 \| Yes \| *** \| \| Naïve vs. EAE + PCB 0.1 mg/kg \| -10.58 \| No \| ns \| \| Naïve vs. EAE + PCB 0.5 mg/kg \| -3.500 \| No \| ns \| \| Naïve vs. EAE + PCB 1 mg/kg \| -0.1131 \| No \| ns \| \| EAE + vehicle vs. EAE + PCB 0.1 mg/kg \| 18.28 \| No \| ns \| \| EAE + vehicle vs. EAE + PCB 0.5 mg/kg \| 25.35 \| Yes \| ** \| \| EAE + vehicle vs. EAE + PCB 1 mg/kg \| 28.74 \| Yes \| *** \| \| EAE + PCB 0.1 mg/kg vs. EAE + PCB 0.5 mg/kg \| 7.077 \| No \| ns \| \| EAE + PCB 0.1 mg/kg vs. EAE + PCB 1 mg/kg \| 10.46 \| No \| ns \| \| EAE + PCB 0.5 mg/kg vs. EAE + PCB 1 mg/kg \| 3.387 \| No \| ns \|   **IL-10**   \| **Kruskal-Wallis test** \|  \| \| --- \| --- \| \| P value \| 0.0026 \| \| Exact or approximate P value? \| Approximate \| \| P value summary \| ** \| \| Do the medians vary signif. (P < 0.05)? \| Yes \| \| Number of groups \| 5 \| \| Kruskal-Wallis statistic \| 16.37 \|  \| **Dunn's multiple comparisons test** \| **Mean rank diff.** \| **Significant?** \| **Summary** \| \| --- \| --- \| --- \| --- \| \| Naïve vs. EAE + vehicle \| -24.83 \| Yes \| ** \| \| Naïve vs. EAE + PCB 0.1 mg/kg \| -11.07 \| No \| ns \| \| Naïve vs. EAE + PCB 0.5 mg/kg \| -3.292 \| No \| ns \| \| Naïve vs. EAE + PCB 1 mg/kg \| -0.6726 \| No \| ns \| \| EAE + vehicle vs. EAE + PCB 0.1 mg/kg \| 13.77 \| No \| ns \| \| EAE + vehicle vs. EAE + PCB 0.5 mg/kg \| 21.54 \| Yes \| * \| \| EAE + vehicle vs. EAE + PCB 1 mg/kg \| 24.16 \| Yes \| ** \| \| EAE + PCB 0.1 mg/kg vs. EAE + PCB 0.5 mg/kg \| 7.774 \| No \| ns \| \| EAE + PCB 0.1 mg/kg vs. EAE + PCB 1 mg/kg \| 10.39 \| No \| ns \| \| EAE + PCB 0.5 mg/kg vs. EAE + PCB 1 mg/kg \| 2.619 \| No \| ns \| |
| **Figure 4.** Histological and immunohistochemical assessment of the PCB effects on myelination and inflammation markers in spinal cords of EAE mice | **LFB/PAS**   \| **Kruskal-Wallis test** \|  \| \| --- \| --- \| \| P value \| <0.0001 \| \| Exact or approximate P value? \| Approximate \| \| P value summary \| **** \| \| Do the medians vary signif. (P < 0.05)? \| Yes \| \| Number of groups \| 4 \| \| Kruskal-Wallis statistic \| 57.56 \|  \| **Dunn's multiple comparisons test** \| **Mean rank diff.** \| **Significant?** \| **Summary** \| \| --- \| --- \| --- \| --- \| \| EAE + vehicle vs. EAE + PCB 0.1 mg/kg \| 7.524 \| No \| ns \| \| EAE + vehicle vs. EAE + PCB 0.5 mg/kg \| 32.15 \| Yes \| ** \| \| EAE + vehicle vs. EAE + PCB 1 mg/kg \| 64.08 \| Yes \| **** \| \| EAE + PCB 0.1 mg/kg vs. EAE + PCB 0.5 mg/kg \| 24.62 \| Yes \| * \| \| EAE + PCB 0.1 mg/kg vs. EAE + PCB 1 mg/kg \| 56.56 \| Yes \| **** \| \| EAE + PCB 0.5 mg/kg vs. EAE + PCB 1 mg/kg \| 31.94 \| Yes \| ** \|   **Mac-3**   \| **Kruskal-Wallis test** \|  \| \| --- \| --- \| \| P value \| <0.0001 \| \| Exact or approximate P value? \| Approximate \| \| P value summary \| **** \| \| Do the medians vary signif. (P < 0.05)? \| Yes \| \| Number of groups \| 4 \| \| Kruskal-Wallis statistic \| 87.48 \|  \| **Dunn's multiple comparisons test** \| **Mean rank diff.** \| **Significant?** \| **Summary** \| \| --- \| --- \| --- \| --- \| \| EAE + vehicle vs. EAE + PCB 0.1 mg/kg \| 61.51 \| Yes \| **** \| \| EAE + vehicle vs. EAE + PCB 0.5 mg/kg \| 64.59 \| Yes \| **** \| \| EAE + vehicle vs. EAE + PCB 1 mg/kg \| 109.9 \| Yes \| **** \| \| EAE + PCB 0.1 mg/kg vs. EAE + PCB 0.5 mg/kg \| 3.077 \| No \| ns \| \| EAE + PCB 0.1 mg/kg vs. EAE + PCB 1 mg/kg \| 48.41 \| Yes \| **** \| \| EAE + PCB 0.5 mg/kg vs. EAE + PCB 1 mg/kg \| 45.33 \| Yes \| **** \|   **CD3**   \| **One-way ANOVA** \| **SS** \| **DF** \| **MS** \| **F (DFn, DFd)** \| **P value** \| \| --- \| --- \| --- \| --- \| --- \| --- \| \| Treatment (between columns) \| 13271392 \| 3 \| 4423797 \| F (3, 86) = 39.34 \| P<0.0001 \| \| Residual (within columns) \| 9670842 \| 86 \| 112452 \|  \|  \| \| Total \| 22942234 \| 89 \|  \|  \|  \|  \| **Newman-Keuls multiple comparisons test** \| **Mean Diff.** \| **Significant?** \| **Summary** \| \| --- \| --- \| --- \| --- \| \| EAE + vehicle vs. EAE + PCB 0.1 mg/kg \| 618.4 \| Yes \| **** \| \| EAE + vehicle vs. EAE + PCB 0.5 mg/kg \| 812.8 \| Yes \| **** \| \| EAE + vehicle vs. EAE + PCB 1 mg/kg \| 1012 \| Yes \| **** \| \| EAE + PCB 0.1 mg/kg vs. EAE + PCB 0.5 mg/kg \| 194.4 \| No \| ns \| \| EAE + PCB 0.1 mg/kg vs. EAE + PCB 1 mg/kg \| 393.5 \| Yes \| *** \| \| EAE + PCB 0.5 mg/kg vs. EAE + PCB 1 mg/kg \| 199.1 \| No \| ns \| |
| **Figure 5.** Immunohistochemical assessment of the PCB effects on oligodendrocyte and neuronal markers in spinal cords of EAE mice | **Olig2**   \| **One-way ANOVA** \| **SS** \| **DF** \| **MS** \| **F (DFn, DFd)** \| **P value** \| \| --- \| --- \| --- \| --- \| --- \| --- \| \| Treatment (between columns) \| 478017 \| 4 \| 119504 \| F (4, 126) = 45.74 \| P<0.0001 \| \| Residual (within columns) \| 329214 \| 126 \| 2613 \|  \|  \| \| Total \| 807231 \| 130 \|  \|  \|  \|  \| **Newman-Keuls multiple comparisons test** \| **Mean Diff.** \| **Significant?** \| **Summary** \| \| --- \| --- \| --- \| --- \| \| Naïve vs. EAE + vehicle \| 107.0 \| Yes \| **** \| \| Naïve vs. EAE + PCB 0.1 mg/kg \| 35.25 \| Yes \| * \| \| Naïve vs. EAE + PCB 0.5 mg/kg \| -13.20 \| No \| ns \| \| Naïve vs. EAE + PCB 1 mg/kg \| -100.8 \| Yes \| **** \| \| EAE + vehicle vs. EAE + PCB 0.1 mg/kg \| -71.75 \| Yes \| **** \| \| EAE + vehicle vs. EAE + PCB 0.5 mg/kg \| -120.2 \| Yes \| **** \| \| EAE + vehicle vs. EAE + PCB 1 mg/kg \| -207.8 \| Yes \| **** \| \| EAE + PCB 0.1 mg/kg vs. EAE + PCB 0.5 mg/kg \| -48.46 \| Yes \| *** \| \| EAE + PCB 0.1 mg/kg vs. EAE + PCB 1 mg/kg \| -136.1 \| Yes \| **** \| \| EAE + PCB 0.5 mg/kg vs. EAE + PCB 1 mg/kg \| -87.63 \| Yes \| **** \|   **TTTP/p25**   \| **One-way ANOVA** \| **SS** \| **DF** \| **MS** \| **F (DFn, DFd)** \| **P value** \| \| --- \| --- \| --- \| --- \| --- \| --- \| \| Treatment (between columns) \| 444382 \| 4 \| 111096 \| F (4, 92) = 17.42 \| P<0.0001 \| \| Residual (within columns) \| 586710 \| 92 \| 6377 \|  \|  \| \| Total \| 1031093 \| 96 \|  \|  \|  \|  \| **Newman-Keuls multiple comparisons test** \| **Mean Diff.** \| **Significant?** \| **Summary** \| \| --- \| --- \| --- \| --- \| \| Naïve vs. EAE + vehicle \| 143.3 \| Yes \| **** \| \| Naïve vs. EAE + PCB 0.1 mg/kg \| 155.4 \| Yes \| **** \| \| Naïve vs. EAE + PCB 0.5 mg/kg \| 52.08 \| No \| ns \| \| Naïve vs. EAE + PCB 1 mg/kg \| -3.118 \| No \| ns \| \| EAE + vehicle vs. EAE + PCB 0.1 mg/kg \| 12.12 \| No \| ns \| \| EAE + vehicle vs. EAE + PCB 0.5 mg/kg \| -91.20 \| Yes \| ** \| \| EAE + vehicle vs. EAE + PCB 1 mg/kg \| -146.4 \| Yes \| **** \| \| EAE + PCB 0.1 mg/kg vs. EAE + PCB 0.5 mg/kg \| -103.3 \| Yes \| *** \| \| EAE + PCB 0.1 mg/kg vs. EAE + PCB 1 mg/kg \| -158.5 \| Yes \| **** \| \| EAE + PCB 0.5 mg/kg vs. EAE + PCB 1 mg/kg \| -55.20 \| No \| ns \|   **APP**   \| **One-way ANOVA** \| **SS** \| **DF** \| **MS** \| **F (DFn, DFd)** \| **P value** \| \| --- \| --- \| --- \| --- \| --- \| --- \| \| Treatment (between columns) \| 232768 \| 3 \| 77589 \| F (3, 159) = 6.967 \| P=0.0002 \| \| Residual (within columns) \| 1770777 \| 159 \| 11137 \|  \|  \| \| Total \| 2003545 \| 162 \|  \|  \|  \|  \| **Newman-Keuls multiple comparisons test** \| **Mean Diff.** \| **Significant?** \| **Summary** \| \| --- \| --- \| --- \| --- \| \| EAE + vehicle vs. EAE + PCB 0.1 mg/kg \| -3.437 \| No \| ns \| \| EAE + vehicle vs. EAE + PCB 0.5 mg/kg \| 56.65 \| Yes \| * \| \| EAE + vehicle vs. EAE + PCB 1 mg/kg \| 87.68 \| Yes \| ** \| \| EAE + PCB 0.1 mg/kg vs. EAE + PCB 0.5 mg/kg \| 60.08 \| Yes \| * \| \| EAE + PCB 0.1 mg/kg vs. EAE + PCB 1 mg/kg \| 91.11 \| Yes \| *** \| \| EAE + PCB 0.5 mg/kg vs. EAE + PCB 1 mg/kg \| 31.03 \| No \| ns \| |
| **Figure 6.** Effect of the treatment with the combination PCB/IFN-β and each compound individually on the clinical progression of EAE mice | **Clinical severity (area under the curve)**  **Prophylactic**   \| **One-way ANOVA** \| **SS** \| **DF** \| **MS** \| **F (DFn, DFd)** \| **P value** \| \| --- \| --- \| --- \| --- \| --- \| --- \| \| Treatment (between columns) \| 2168 \| 3 \| 722.5 \| F (3, 33) = 15.90 \| P<0.0001 \| \| Residual (within columns) \| 1500 \| 33 \| 45.44 \|  \|  \| \| Total \| 3667 \| 36 \|  \|  \|  \|  \| **Newman-Keuls multiple comparisons test** \| **Mean Diff.** \| **Significant?** \| **Summary** \| \| --- \| --- \| --- \| --- \| \| EAE + vehicle vs. EAE + IFN-β 5000 U \| 9.081 \| Yes \| ** \| \| EAE + vehicle vs. EAE + PCB 1 mg/kg \| 16.56 \| Yes \| **** \| \| EAE + vehicle vs. EAE + PCB 1 mg/kg / IFN-β 5000 U \| 20.25 \| Yes \| **** \| \| EAE + IFN-β 5000 U vs. EAE + PCB 1 mg/kg \| 7.475 \| Yes \| * \| \| EAE + IFN-β 5000 U vs. EAE + PCB 1 mg/kg / IFN-β 5000 U \| 11.17 \| Yes \| ** \| \| EAE + PCB 1 mg/kg vs. EAE + PCB 1 mg/kg / IFN-β 5000 U \| 3.694 \| No \| ns \|   **Late therapeutic**   \| **One-way ANOVA** \| **SS** \| **DF** \| **MS** \| **F (DFn, DFd)** \| **P value** \| \| --- \| --- \| --- \| --- \| --- \| --- \| \| Treatment (between columns) \| 84.07 \| 3 \| 28.02 \| F (3, 34) = 0.4995 \| P=0.6851 \| \| Residual (within columns) \| 1908 \| 34 \| 56.11 \|  \|  \| \| Total \| 1992 \| 37 \|  \|  \|  \|  \| **Newman-Keuls multiple comparisons test** \| **Mean Diff.** \| **Significant?** \| **Summary** \| \| --- \| --- \| --- \| --- \| \| EAE + vehicle vs. EAE + IFN-β 5000 U \| 1.736 \| No \| ns \| \| EAE + vehicle vs. EAE + PCB 1 mg/kg \| 4.025 \| No \| ns \| \| EAE + vehicle vs. EAE + PCB 1 mg/kg / IFN-β 5000 U \| 2.542 \| No \| ns \| \| EAE + IFN-β 5000 U vs. EAE + PCB 1 mg/kg \| 2.289 \| No \| ns \| \| EAE + IFN-β 5000 U vs. EAE + PCB 1 mg/kg / IFN-β 5000 U \| 0.8056 \| No \| ns \| \| EAE + PCB 1 mg/kg vs. EAE + PCB 1 mg/kg / IFN-β 5000 U \| -1.483 \| No \| ns \| |
| **Figure 7.** Effect of the treatment with the combination PCB/IFN-β and each compound individually  on the cerebral expression of cytokines (protein levels) and of demyelinating / remyelinating genes (mRNA levels), as well as on the Treg levels in spleen of EAE mice | **IL-17A**   \| **Kruskal-Wallis test** \|  \| \| --- \| --- \| \| P value \| 0.0013 \| \| Exact or approximate P value? \| Approximate \| \| P value summary \| ** \| \| Do the medians vary signif. (P < 0.05)? \| Yes \| \| Number of groups \| 5 \| \| Kruskal-Wallis statistic \| 17.93 \|  \| **Dunn's multiple comparisons test** \| **Mean rank diff.** \| **Significant?** \| **Summary** \| \| --- \| --- \| --- \| --- \| \| EAE + vehicle vs. Naïve \| 18.67 \| Yes \| *** \| \| EAE + vehicle vs. EAE + IFN-β 5000 U \| 13.87 \| Yes \| * \| \| EAE + vehicle vs. EAE + PCB 1 mg/kg \| 8.000 \| No \| ns \| \| EAE + vehicle vs. EAE + PCB 1 mg/kg / IFN-β 5000U \| 13.87 \| Yes \| * \|   **IL-6**   \| **Kruskal-Wallis test** \|  \| \| --- \| --- \| \| P value \| 0.0012 \| \| Exact or approximate P value? \| Approximate \| \| P value summary \| ** \| \| Do the medians vary signif. (P < 0.05)? \| Yes \| \| Number of groups \| 5 \| \| Kruskal-Wallis statistic \| 18.09 \|  \| **Dunn's multiple comparisons test** \| **Mean rank diff.** \| **Significant?** \| **Summary** \| \| --- \| --- \| --- \| --- \| \| EAE + vehicle vs. Naïve \| 18.01 \| Yes \| *** \| \| EAE + vehicle vs. EAE + IFN-β 5000 U \| 13.10 \| Yes \| * \| \| EAE + vehicle vs. EAE + PCB 1 mg/kg \| 8.429 \| No \| ns \| \| EAE + vehicle vs. EAE + PCB 1 mg/kg / IFN-β 5000U \| 16.30 \| Yes \| ** \|   **IL-10**   \| **One-way ANOVA** \| **SS** \| **DF** \| **MS** \| **F (DFn, DFd)** \| **P value** \| \| --- \| --- \| --- \| --- \| --- \| --- \| \| Treatment (between columns) \| 305439 \| 4 \| 76360 \| F (4, 25) = 6.085 \| P=0.0015 \| \| Residual (within columns) \| 313732 \| 25 \| 12549 \|  \|  \| \| Total \| 619171 \| 29 \|  \|  \|  \|  \| **Newman-Keuls multiple comparisons test** \| **Mean Diff.** \| **Significant?** \| **Summary** \| \| --- \| --- \| --- \| --- \| \| Naïve vs. EAE + vehicle \| -216.6 \| Yes \| ** \| \| Naïve vs. EAE + IFN-β 5000 U \| -1.209 \| No \| ns \| \| Naïve vs. EAE + PCB 1 mg/kg \| -39.68 \| No \| ns \| \| Naïve vs. EAE + PCB 1 mg/kg / IFN-β 5000 U \| 77.86 \| No \| ns \| \| EAE + vehicle vs. EAE + IFN-β 5000 U \| 215.4 \| Yes \| ** \| \| EAE + vehicle vs. EAE + PCB 1 mg/kg \| 176.9 \| Yes \| ** \| \| EAE + vehicle vs. EAE + PCB 1 mg/kg / IFN-β 5000 U \| 294.5 \| Yes \| ** \| \| EAE + IFN-β 5000 U vs. EAE + PCB 1 mg/kg \| -38.47 \| No \| ns \| \| EAE + IFN-β 5000 U vs. EAE + PCB 1 mg/kg / IFN-β 5000 U \| 79.07 \| No \| ns \| \| EAE + PCB 1 mg/kg vs. EAE + PCB 1 mg/kg / IFN-β 5000 U \| 117.5 \| No \| ns \|   **Foxp3 (MFI)**   \| **One-way ANOVA** \| **SS** \| **DF** \| **MS** \| **F (DFn, DFd)** \| **P value** \| \| --- \| --- \| --- \| --- \| --- \| --- \| \| Treatment (between columns) \| 126.4 \| 4 \| 31.60 \| F (4, 24) = 2.803 \| P=0.0485 \| \| Residual (within columns) \| 270.6 \| 24 \| 11.27 \|  \|  \| \| Total \| 397.0 \| 28 \|  \|  \|  \|  \| **Newman-Keuls multiple comparisons test** \| **Mean Diff.** \| **Significant?** \| **Summary** \| \| --- \| --- \| --- \| --- \| \| Naïve vs. EAE + vehicle \| -5.131 \| No \| ns \| \| Naïve vs. EAE + IFN-β 5000 U \| -3.395 \| No \| ns \| \| Naïve vs. EAE + PCB 1 mg/kg \| -4.436 \| No \| ns \| \| Naïve vs. EAE + PCB 1 mg/kg / IFN-β 5000 U \| -6.612 \| Yes \| * \| \| EAE + vehicle vs. EAE + IFN-β 5000 U \| 1.736 \| No \| ns \| \| EAE + vehicle vs. EAE + PCB 1 mg/kg \| 0.6945 \| No \| ns \| \| EAE + vehicle vs. EAE + PCB 1 mg/kg / IFN-β 5000 U \| -1.481 \| No \| ns \| \| EAE + IFN-β 5000 U vs. EAE + PCB 1 mg/kg \| -1.042 \| No \| ns \| \| EAE + IFN-β 5000 U vs. EAE + PCB 1 mg/kg / IFN-β 5000 U \| -3.217 \| No \| ns \| \| EAE + PCB 1 mg/kg vs. EAE + PCB 1 mg/kg / IFN-β 5000 U \| -2.176 \| No \| ns \| |
