# Supplementary figures and images for: Anti-inflammatory mechanisms and pharmacological actions of phycocyanobilin in a mouse model of experimental autoimmune encephalomyelitis: A therapeutic promise for multiple sclerosis
Source: Front Immunol. 2022 Nov 3;13:1036200. doi: 10.3389/fimmu.2022.1036200 (PMC9669316; doi:10.3389/fimmu.2022.1036200)

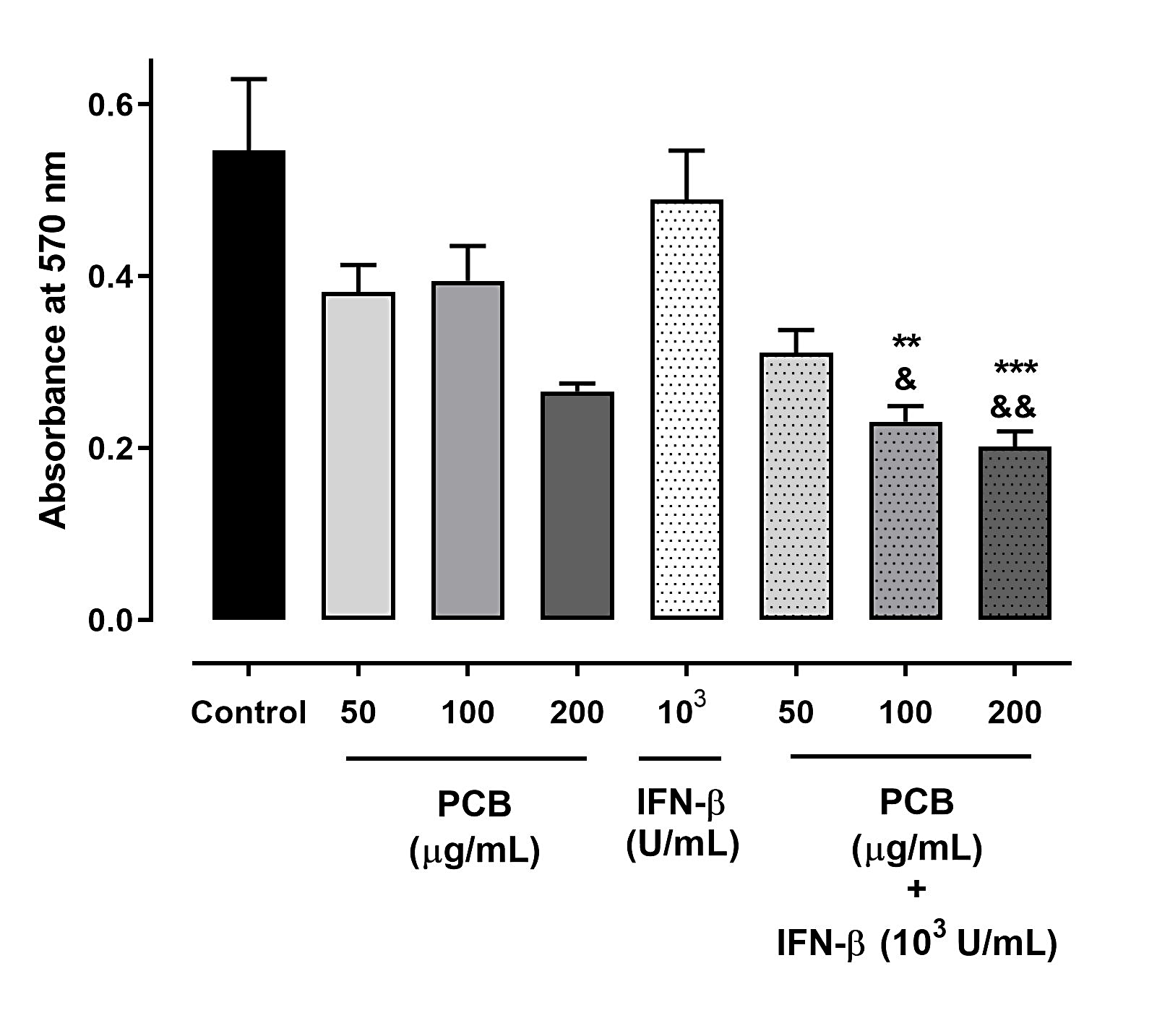

Supplement: Supplementary Figure 1 — Effects of the combination PCB + IFN-β on rat encephalitogenic TMBP-GFP cells. Effect on the proliferation: TMBP-GFP cells (4 x 10^4^ cells/well) were cultured for 48 h in different treatment conditions in 96-well plate with the presence of irradiated rat thymocytes (5000 rad, 106 cells/well). Complete DMEM/1% rat serum with 10 µg/mL MBP was used. Immediately after, 0.5 mg/mL MTT was added to the medium and cultured for another 4 h. After medium removal, the resulting formazan was solubilized and its absorbance was measured at 570 nm. Data is presented as mean ± S.E.M. Ampersand (&) and asterisk (*) indicate significant differences in comparison to 103 U/mL IFN-β and control, respectively (**p<0.01, ***p<0.001 vs control; &p<0.05, &&p <0.01 vs IFN-β, according to Kruskal-Wallis + Dunn’s tests). [file Image_1.tif]

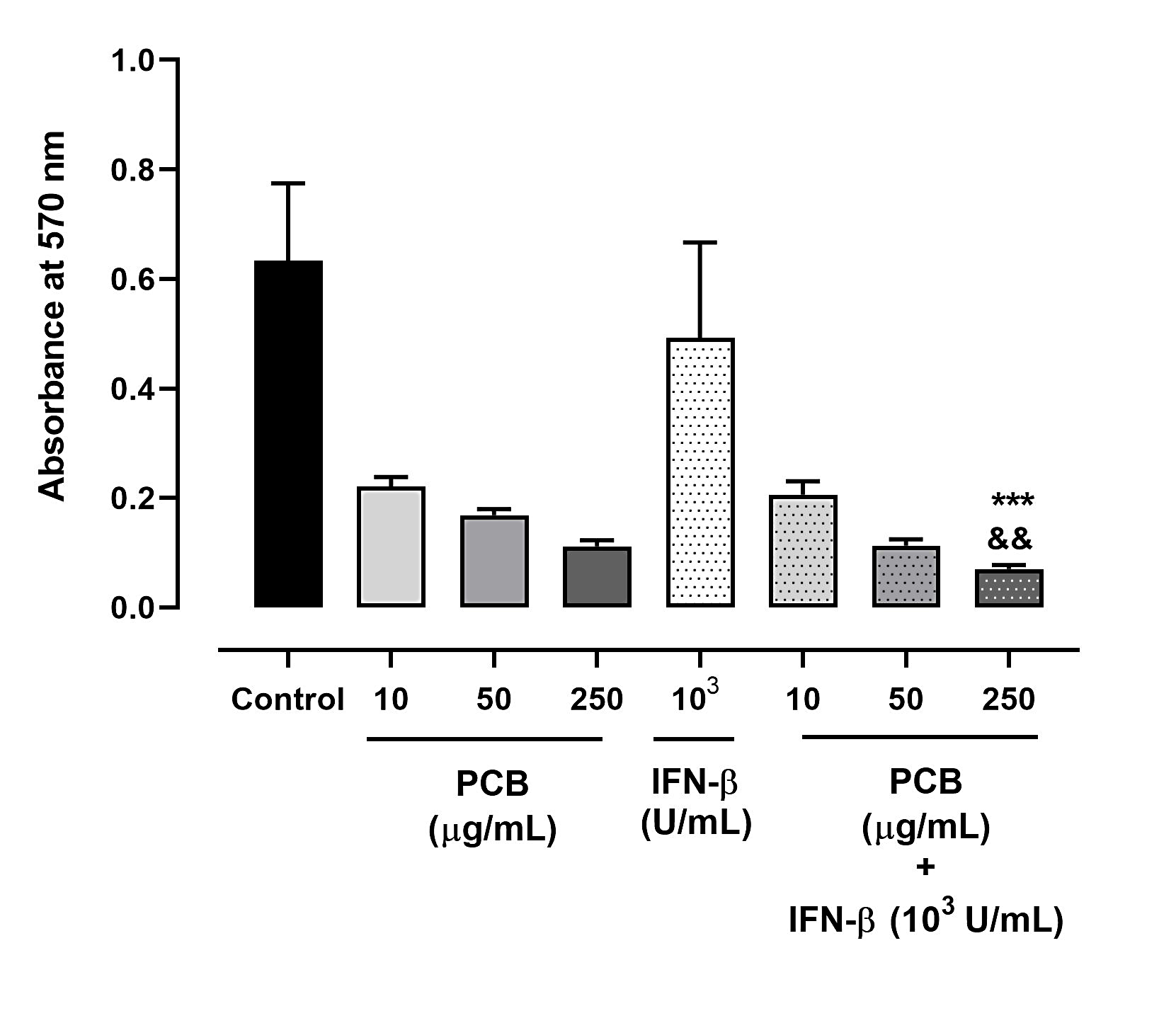

Supplement: Supplementary Figure 2 — Effects of the combination PCB + IFN-β on the proliferation of CD4+ T cells from 2D2 mice. PCB was incubated with purified CD4+ T cells at different concentrations in the presence of absence of 5 x 10^3^ U/mL IFN-β. A seeding density of 2 x 10^5^ cells/well was used for the co-stimulation of CD4+ T cells with anti-CD3 and anti-CD28 at 2 µg/mL in 96-well plate. Two days later, CD4+ T cells were incubated with IL-2 at 10 ng/mL for another 24 h. Then, MTT assay was performed and its cellular reduction was measured by the absorbance at 570 nm. Positive and negative controls indicate the absence of the drugs in the medium or no incubation with anti-CD3/anti-CD28/IL-2/drugs, respectively. Data is presented as mean ± S.E.M. Ampersand (&) and asterisk (*) indicate significant differences in comparison to 103 U/mL IFN-β and control, respectively (***p<0.001 vs control; &&p <0.01 vs IFN-β, according to Kruskal-Wallis + Dunn’s tests). [file Image_2.tif]
